# Morphology of obligate ectosymbionts reveals *Paralaxus* gen. nov.: A new circumtropical genus of marine stilbonematine nematodes

**DOI:** 10.1111/zsc.12399

**Published:** 2020-02-20

**Authors:** Florian Scharhauser, Judith Zimmermann, Jörg A. Ott, Nikolaus Leisch, Harald R. Gruber‐Vodicka

**Affiliations:** ^1^ Department of Limnology and Bio‐Oceanography University of Vienna Vienna Austria; ^2^ Max Planck Institute for Marine Microbiology Bremen Germany

**Keywords:** 16S rRNA, 18S rRNA, cytochrome c oxidase subunit I, ectosymbionts, molecular phylogeny, *Paralaxus*, systematics, thiotrophic symbiosis

## Abstract

Stilbonematinae are a subfamily of conspicuous marine nematodes, distinguished by a coat of sulphur‐oxidizing bacterial ectosymbionts on their cuticle. As most nematodes, the worm hosts have a relatively simple anatomy and few taxonomically informative characters, and this has resulted in numerous taxonomic reassignments and synonymizations. Recent studies using a combination of morphological and molecular traits have helped to improve the taxonomy of Stilbonematinae but also raised questions on the validity of several genera. Here, we describe a new circumtropically distributed genus *Paralaxus* (Stilbonematinae) with three species: *Paralaxus cocos* sp. nov., *P. bermudensis* sp. nov. and *P. columbae* sp. nov. We used single worm metagenomes to generate host 18S rRNA and cytochrome c oxidase I (COI) as well as symbiont 16S rRNA gene sequences. Intriguingly, COI alignments and primer matching analyses suggest that the COI is not suitable for PCR‐based barcoding approaches in Stilbonematinae as the genera have a highly diverse base composition and no conserved primer sites. The phylogenetic analyses of all three gene sets, however, confirm the morphological assignments and support the erection of the new genus *Paralaxus* as well as corroborate the status of the other stilbonematine genera. *Paralaxus* most closely resembles the stilbonematine genus *Laxus* in overlapping sets of diagnostic features but can be distinguished from *Laxus* by the morphology of the genus‐specific symbiont coat. Our re‐analyses of key parameters of the symbiont coat morphology as character for all Stilbonematinae genera show that with amended descriptions, including the coat, highly reliable genus assignments can be obtained.

## 1 INTRODUCTION

The identification of many nematode genera and species is difficult based on morphological characters alone (Derycke et al., 2008; de‐León & Nadler, 2010; Palomares‐Rius, Cantalapiedra‐Navarrete, & Castillo, 2014; Sudhaus & Kiontke, 2007). A prime example for this problem is the Stilbonematinae, a subfamily of the Desmodoridae that have experienced several changes in the classification of species and even genera in the past (Ott, Gruber‐Vodicka, Leisch, & Zimmermann, 2014; Tchesunov, 2013). The exclusively marine Stilbonematinae are common members of the interstitial meiofauna in sheltered intertidal and subtidal porous sediments and have been found worldwide (Ott, Bright, & Bulgheresi, 2004; Tchesunov, 2013). Their diversity and abundances are highest in subtropical and tropical shallow‐water sands, but some species were also described from higher latitudes (e.g. Gerlach, 1950; Platt & Zhang, 1982; Riemann, Thiermann, & Bock, 2003; Tchesunov, Ingels, & Popova, 2012; Wieser, 1960), deep‐sea sediments (Leduc, 2013; Tchesunov et al., 2012; Van Gaever, Vanreusel, Hughes, Bett, & Kiriakoulakis, 2004), near shallow hydrothermal vents (Kamenev, Fadeev, Selin, Tarasov, & Malakhov, 1993; Thiermann, Koumanidis, Hughes, & Giere, 1997) and methane seeps (Dando, Hughes, & Thiermann, 1995).

To date, a total of 10 different stilbonematine nematode genera and more than 50 different species are described (reviewed by Armenteros, Ruiz‐Abierno, & Decraemer, 2014; Leduc, 2013; Leduc & Sinniger, 2018; Ott, Gruber‐Vodicka, et al., 2014; Ott Leisch & Gruber‐Vodicka, 2014; Tchesunov, 2013). Most species and genera still lack molecular data, but recent taxonomic work has started to integrate molecular data using the 18S rRNA gene (18S) (Armenteros, Rojas‐Corzo, et al., 2014; Armenteros, Ruiz‐Abierno, et al., 2014; Leduc & Sinniger, 2018; Leduc & Zhao, 2016; Ott, Gruber‐Vodicka, et al., 2014) and the mitochondrial cytochrome c oxidase subunit I (COI) gene (Armenteros, Rojas‐Corzo, et al., 2014). The phylogenetic signal of the two marker genes was, however, not consistent and called morphological genus and species identifications into question (Armenteros, Ruiz‐Abierno, et al., 2014). While the monophyly of the subfamily Stilbonematinae has strong support based on 18S data (Kampfer, Sturmbauer, & Ott, 1998; Bayer et al., 2009; van Megen et al., 2009; Ott, Gruber‐Vodicka, et al., 2014 and Ott, Leisch, et al., 2014; Leduc & Zhao, 2016), it was questioned in a recent study by Armenteros, Ruiz‐Abierno, et al. (2014) which used both 18S and COI.

A trait that unites all stilbonematine nematodes is the conspicuous ectosymbiosis with sulphur‐oxidizing *Gammaproteobacteria* that cover major parts of the cuticle (reviewed by Ott et al., 2004). Stilbonematine symbionts have diverse morphologies, including rod, coccus, coccobacillus, filament, corn kernel or crescent shapes, and each host species harbours a single symbiont morphotype (Ott & Novak, 1989; Polz, Felbeck, Novak, Nebelsick, & Ott, 1992; Polz et al., 1994; Ott et al., 2004; Bayer et al., 2009; Pende et al., 2014; Ott, Gruber‐Vodicka, et al., 2014). In phylogenetic analyses of the symbiont 16S rRNA gene (16S) sequences, all stilbonematine symbionts fall into a monophyletic clade of *Gammaproteobacteria* related to *Chromatiaceae*, which has been described as *Candidatus* Thiosymbion (from here on ‘Thiosymbion’) (Bayer et al., 2009; Bulgheresi et al., 2011; Heindl et al., 2011; Zimmermann et al., 2016). Based on phylogenetic analyses of host 18S and symbiont 16S sequences closely related stilbonematine nematode species of the same genus consistently harbour closely related and genus‐specific ‘Thiosymbion’ symbionts (Ott et al., 2004; Zimmermann et al., 2016). ‘Thiosymbion’ encompasses not only the ectosymbionts of stilbonematine nematodes, but also the primary symbionts of gutless phallodriline oligochaetes and siphonolaimid nematodes (Zimmermann et al., 2016). Comparative analyses of stilbonematine nematodes and their symbionts showed a high degree of phylogenetic congruence that indicates a long‐term co‐diversification between ‘Thiosymbion’ and the Stilbonematinae (Zimmermann et al., 2016).

Recent 18S‐based phylogenetic analyses suggested a novel, morphologically uncharacterized stilbonematine genus from the Caribbean Sea (Belize) and the Australian Great Barrier Reef (Heron Island; Zimmermann et al., 2016). The genus level of this clade was supported by the distinct 16S sequences of their symbionts that also formed a new phylogenetic clade in the study. Here, we combine morphological and molecular data to describe this recently detected host clade as *Paralaxus* gen. nov. We use single worm metagenomes to provide host 18S and COI gene sequences as well as symbiont 16S sequences from nine *Paralaxus* gen. nov. specimens and 20 additional specimens covering six stilbonematine genera. We place *Paralaxus* gen. nov. in a unified taxonomy of Stilbonematinae and formally describe the new genus *Paralaxus* with a Caribbean type species *P. cocos* sp. nov*.* In addition, we describe two new species, *P. bermudensis* sp. nov. and *P. columbae* sp. nov. from Bermuda and Florida, and report on members of this new genus from localities in the Western and Central Pacific Ocean. While characterizing the key anatomical features of *Paralaxus* gen. nov., we identified the high taxonomic value of its symbiont coat. We extend this concept and evaluate coat and symbiont morphology as additional traits for all stilbonematine genera.

In addition, we substantially improve the molecular dataset of Stilbonematinae full‐length 18S and COI marker gene sequences to answer the following questions: (a) Are Stilbonematinae monophyletic? (b) At which taxonomic level are 18S and COI valuable phylogenetic markers for Stilbonematinae? (c) Can we find suitable COI PCR priming sites that are conserved across the subfamily?

## 2 MATERIAL AND METHODS

### 2.1 Meiofauna collection, preparation and microscopy

**Figure 1:**
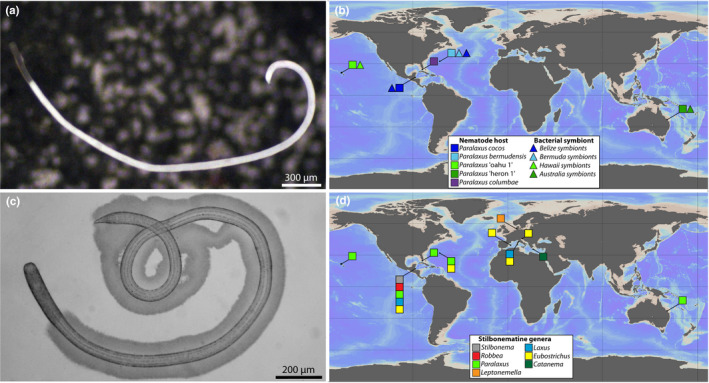
*Paralaxus* gen. nov. habitus and sampling sites of stilbonematine nematodes. (a) Micrograph of *Paralaxus bermudensis* sp. nov. The symbiont‐free anterior region is clearly visible compared to the densely coated white body. (b) Sampling locations of *Paralaxus* species included in this study. (c) Juvenile specimen of *Paralaxus columbae* sp. nov. with thick symbiotic coat and bacteria‐free head region. (d) Sampling locations of other stilbonematine nematode species included in this study. Specimens for this study were sampled from shallow‐water sandy sediments in the Caribbean Sea, the Atlantic and the Pacific Ocean as well as the North and Mediterranean Sea. For details, see Table S1 (Supporting information)

Sediment samples were collected in the Caribbean Sea (Belize), the Western Atlantic Ocean (Florida and Bermuda), the Western and Central Pacific Ocean (Australia and Hawaii) as well as the North and Mediterranean Sea (Figure 1 and Figure S1) using cores or buckets. Nematodes were extracted by decantation through a 64‐µm mesh sieve and sorted live under a dissecting microscope. Specimens were fixed in 4% formaldehyde for taxonomic preparations or in 2.5% glutaraldehyde in 0.1 M sodium cacodylate buffer and were postfixed in 2% OsO_4_ (for scanning electron microscopy, *SEM*) and stored at 4°C until further analyses. Before fixation, live specimens for sequencing were photographically vouchered in the field; *that is,* micrographs were taken of the full specimen mounted in seawater, capturing the head region with mouth, amphids and pharynx, the anal and tail region, the symbiont coat, and male and female reproductive organs. After imaging, specimens were washed in 0.2 µm filtered seawater and immediately fixed in either RNAlater® stabilization solution (Ambion, Foster City, US), 70% ethanol or pure methanol and stored at 4°C until the molecular analyses.

For detailed light microscopy, the formaldehyde or glutaraldehyde‐fixed specimens were transferred into glycerol: water 1:9, slowly evaporated and finally mounted in pure glycerol. Drawings were made using a camera lucida on a Diavar microscope (Reichert, Vienna, Austria). Nomarsky interference contrast photographs were taken on a Polyvar (Reichert, Vienna, Austria). Specimens for *SEM* were critical point dried and coated with palladium using a JEOL JFC‐2300HR sputter coater (JEOL Ltd., Akishima, Japan) and examined on a JEOL IT 300 (JEOL Ltd., Akishima, Japan) at high vacuum mode.

### 2.2 DNA extraction, marker gene amplification and metagenome sequencing

DNA from single nematodes including their attached ectosymbionts was extracted with the DNAeasy Blood and Tissue Micro Kit (Qiagen, Hilden, Germany) following manufacturer's instructions with two amendments to the protocol—the proteinase K digestion was extended to at least 24 hr and the elution step was performed twice with 20 µl of elution buffer. DNA concentrations were quantified using a Qubit 2.0 and the high sensitivity assay (Life Technologies, Carlsbad, US).

To characterize nematode hosts prior to metagenome sequencing, we used both the ribosomal 18S and mitochondrial COI marker genes for PCR‐based screenings. To amplify an approximately 600‐bp‐long fragment of the nematode 18S, we designed a nematode‐specific primer pair: 3FNem 5′‐GTTCGACTCCGGAGAGGGA‐3′ and 5R 5′‐CTTGGCAAATGCTTTCGC‐3′. Amplification of the mitochondrial COI gene of several stilbonematine nematode genera including *Paralaxus* was conducted using the published primer pair 1490F 5′‐GGTCAACAAATCATAAAGATATTGG‐3′ and 2198R 5′‐TAAACTTCAGGGTGACCAAAAATCA‐3′ (Folmer, Black, Hoeh, Lutz, & Vrijenhoek, 1994). For 18S PCRs, we used the Phusion® DNA polymerase, HF buffer (Finnzymes, Finland) and the following cycling conditions: 95°C for 5 min, followed by 38 cycles of 95°C for 1 min, 55°C for 1.5 min, 72°C for 2 min and followed by 72°C for 10 min. COI PCRs were conducted using the TaKaRa Taq™ DNA polymerase, 10× reaction buffer (Takara Bio Inc., Japan) and the following cycling conditions: 5 min at 95°C, then 36 cycles of 1 min at 95°C, 1.5 min at 42°C, and 2 min at 72°C, followed by 72°C for 10 min. While the 18S amplifications were successful for all nematode specimens, we could not obtain a single PCR product for the COI amplifications of any stilbonematine nematode specimen. In contrast, DNA extracts from *Lamellibrachia* tubeworms (Siboglinidae, Annelida) that were used as positive control always resulted in COI PCR products of the expected size.

We performed single specimen shotgun metagenome sequencing to generate data from the host nuclear genome, host mitochondria and symbionts using Illumina short‐read technology. For Illumina sequencing, 1–5 ng DNA per specimen were used for an Ovation Ultralow Library Systems kit (NuGEN Technologies, San Carlos, US). Illumina library preparation including size‐selection on an agarose gel was performed at the Max Planck Genome Centre in Cologne, Germany. 2 × 100 bp paired‐end reads were sequenced on an Illumina HiSeq3000 (San Diego, US) for each individual (8–25 million reads each). We intended to extract the triple gene sets (symbiont 16S, host 18S and COI) for all specimens but differing assembly qualities due to the very low DNA input did not allow us to retrieve all marker genes for each worm (Table S1). Host 18S and symbiont 16S gene sequences were generated from the raw reads with phyloFlash (Gruber‐Vodicka, Pruesse, & Seah, 2017, https://github.com/HRGV/phyloFlash). The COI was assembled using the following approach: In brief, after removing adapters and low‐quality bases (*Q* < 2) with bbduk (Bushnell, 2014), we retained all reads with a minimal length of 36 bp after quality trimming and conducted combined host and symbiont draft genome assemblies for all samples with SPAdes 3.1–3.9 (Bankevich et al., 2012). The contig containing the COI gene was then extracted from each assembly using BLAST 2.26+ searches with the available Stilbonematinae COI sequences as implemented in the Geneious software v. 11.1 (54) (Biomatters, New Zealand). Full‐length COI genes were then predicted from the contigs using the Geneious gene prediction tool.

### 2.3 COI compositional analyses and primer matching

All compositional and primer matching analyses were conducted with the 27 full‐length stilbonematine nematode COI sequences we derived from the metagenomics data. Nucleotide composition was analysed in Geneious. A primer mismatch analysis of the two COI primer sets: 1490F: 5′‐GGT CAA CAA ATC ATA AGA TAT TGG‐3′ and 2198R: 5′‐TAA ACT TCA GGG TGA CCA AAA AAT CA‐3′ (Folmer et al., 1994) and JB3F: 5′‐TTT TTT GGG CAT CCT GAG GTT TAT‐3′ and JB5 R: 5′‐AGC ACC TAA ACT TAA AAC ATA ATG AAA ATG‐3′ used by Armenteros, Rojas‐Corzo, et al. (2014), Armenteros, Ruiz‐Abierno, et al. (2014) was performed using the MOTIF search option integrated in Geneious, with a maximum mismatch rate range from 1 to 7 for every primer. The mismatches were mapped on a MAFFT v.7 (Katoh & Standley, 2013) alignment. The design of a new stilbonematine‐specific COI primer set was conducted with Primer 3 (Untergasser et al., 2012). In brief, the consensus sequence of our full‐length COI MAFFT alignment (1–1,577 bp) was screened for a stilbonematine nematodes‐specific primer set by applying Tm calculation after SantaLucia (1998) and the following parameters: primer size range 18–27 bp, melting temperature (Tm) range 57–63°C and a GC percentage range 20–80. In addition, separate searches were conducted for the regions 1–750 bp and 750–1,577 bp to facilitate the detection of primer pairs that cover similar ranges as the existing primer sets that only amplify parts of the gene.

### 2.4 Phylogenetic analyses of host and symbiont genes

For the host 18S‐based phylogenetic reconstruction, we used 23 full‐length sequences extracted from the single worm metagenomes together with 55 previously published and non‐chimeric stilbonematine nematode 18S sequences longer than 1,300 bp available in GenBank. All published non‐stilbonematine nematode sequences of the order Desmodorida and Chromadorida sequences longer than 1,300 bp available in 10/2018 served as outgroup.

In an extended 18S dataset with a total of 250 sequences, we included eight partial 18S sequences of five stilbonematine species from Armenteros, Ruiz‐Abierno, et al. (2014) and all non‐chimeric 18S sequences of Desmodorida and Chromadorida with a minimum length of 700 bp available in GenBank.

For the host COI phylogeny, we constructed a COI matrix using the 27 full‐length stilbonematine COI sequences we extracted from the metagenomics data of nine *Paralaxus* specimens, together with sequences of 18 specimens from six different stilbonematine genera (*Catanema, Eubostrichus, Laxus, Leptonemella, Robbea and Stilbonema*). We added the full‐length COI sequence from *Baylisascaris procyonis* (JF951366) as outgroup. All 28 nucleotide COI sequences were translated into protein sequences in Geneious using the invertebrate mitochondrial translation table 5. In an extended COI matrix, we included partial COI sequences of the five species from Armenteros, Ruiz‐Abierno, et al. (2014) that were also included in the extended 18S dataset. Due to the high morphological similarity between *Paralaxus cocos* sp. nov. and *Leptonemella brevipharynx* (Armenteros, Ruiz‐Abierno, et al., 2014), the partial COI sequence of *L. brevipharynx* was also included in the analysis.

For the symbiont phylogenetic reconstruction, we used the 21 new 16S sequences that we extracted from the metagenomes as well as the 54 ‘Thiosymbion’ 16S gene sequences available in GenBank. We also included sequences of closely related uncultured *Gammaproteobacteria* (JF344100, JF344607, JF344324) and used five cultured free‐living gammaproteobacterial sulphur‐oxidizing bacteria from the *Chromatiaceae * (*Allochromatium vinosum* strain DSM180, *Marichromatium purpuratum* 984, *Thiorhodococcus drewsii* AZ1, *Thiocapsa marina* 5811, *Thiorhodovibrio* sp. 970) as an outgroup.

The host and symbiont ribosomal rRNA sequences were independently aligned using MAFFT v7 (Katoh & Standley, 2013) with the Q‐INS‐I mode (Katoh & Toh, 2008) as it considers the predicted secondary structure of the RNA. All COI sequences were translated into AA sequences and aligned in MAFFT v7 with the E‐INS‐i mode. The optimal substitution model for each alignment was assessed using ModelFinder (Kalyaanamoorthy, Minh, Wong, Haeseler, & Jermiin, 2017). The mtZOA + F + G4 model was the best fit for the COI alignment, TIM3e + I + G4 for the 18S alignment and TIM + F + I + G4 for the 16S alignment.

Phylogenetic trees were reconstructed using the maximum likelihood‐based software IQTREE (Nguyen, Schmidt, Haeseler, & Minh, 2015) utilizing the Ultrafast Bootstrap Approximation UFBoot (Minh, Nguyen, & Haeseler, 2013) to assess node stability (10,000 bootstrap runs). In addition to maximum likelihood, support values were generated using approximate Bayes (aBayes) (Anisimova, Gil, Dufayard, Dessimoz, & Gascuel, 2011) and SH‐aLRT analyses (Guindon et al., 2010).

## 3 RESULTS

### 3.1 Descriptions

We analysed 33 *Paralaxus* gen. nov. specimens from seven locations in the Pacific, the Atlantic and the Caribbean using light and electron microscopy as well as molecular analyses based on marker genes extracted from single specimen metagenomes.

***Paralaxus*** gen. nov.

ZooBank registration (http://zoobank.org): urn:lsid:zoobank.org:act:417691DB‐5EE3‐49B7‐B838‐1BBF1569235F.

GenBank entries for 18S rRNA and COI sequences: see Table S1.

**Figure 2:**
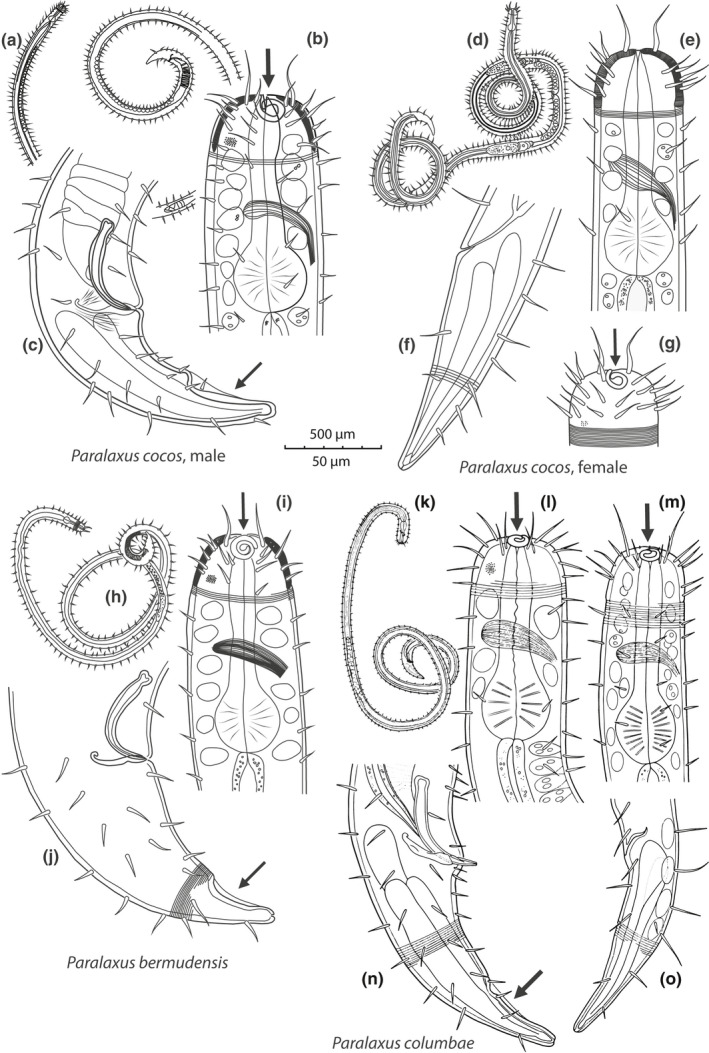
Drawings of three different *Paralaxus* species comparing their defining characters. (a‐c) *Paralaxus cocos* sp. nov. male, (a) total view, (b) anterior end (c) posterior end (d‐g) *P. cocos* sp. nov. female, (d) total view (e) anterior end, optical section (f) posterior end of female (g) anterior surface view (h–j) *P. bermudensis* nov. spec. male (h) total view (i) anterior end (j) posterior end (k–o) *P. columbae* nov. spec. male and female. (k) total view of male paratype, (l) anterior end of male, (m) anterior end of female (n) posterior end of male (o) posterior end of female. Camera lucida drawings. Arrows point to amphidial fovea and velum on male tail

**Figure 3:**
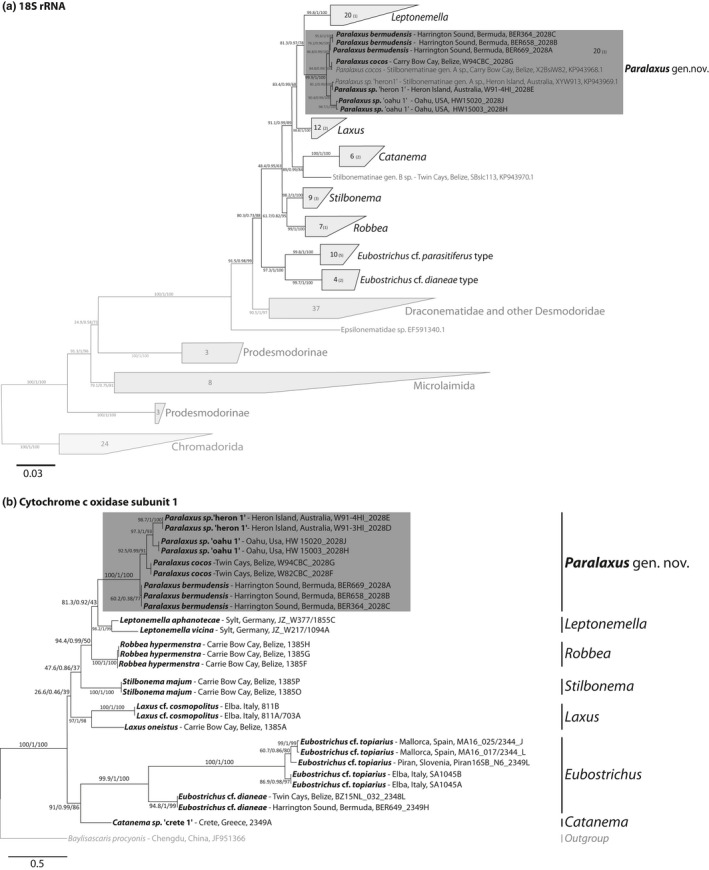
Phylogenetic relationship of *Paralaxus* species with other stilbonematine nematodes based on the 18S rRNA (a) and cytochrome c oxidase subunit I (COI) genes (b). The trees were calculated using IQTree. Support values are given in the following order: SH‐aLRT support (%)/aBayes support/ultrafast bootstrap support (%). Species described in this study are highlighted in bold. Provisional working names for undescribed genera or species are given in quotes. The scale bar represents average nucleotide substitutions per site. Accession numbers for sequences from this study are listed in Table S1

We erect a new genus for this clade with the following characters: cephalic capsule without block layer (Figure 2b, e, i, l, m); amphidial fovea spiral, no sexual dimorphism in shape of fovea (Figure 2b, g, i, l, m); male tail with velum (Figure 2c, j, n); symbiotic bacteria coccobacilli, arranged as multilayered coat covering body except for anterior and posterior end (Figure 1a, b). All *Paralaxus* sequences form statistically supported clades in phylogenetic analyses of the 18S as well as the COI datasets (Figure 3a, b). The specimens for each *Paralaxus* species clustered together in both gene sets and clearly separated each *Paralaxus* species from each other (Figure 3a, b).

We describe three new species and report on two additional ones.

***Paralaxus cocos*** sp. nov.

ZooBank registration (http://zoobank.org): urn:lsid:zoobank.org:act A659918E‐FD88‐4584–8115‐CD4C396AE400.

GenBank entries for 18S rRNA and COI sequences: see Table S1.

Species from Belize, Central America, can be identified by an amphidial fovea with one and a half turns and the bulbus occupies 32%–35% of the pharynx length. The velum of male specimens makes up 43%–47% of the tail length and the spicula are moderately arcuate (Figure 2a‐g).

***Paralaxus bermudensis*** sp. nov.

ZooBank registration (http://zoobank.org): urn:lsid:zoobank.org:act: 8697C6A0‐3508–4833‐81DC‐D2A86B0B00AC.

GenBank entries for 18S rRNA and COI sequences: see Table S1.

Species from Bermuda can be identified by an amphidial fovea with two turns and the bulbus occupies 30%–32% of the pharynx length. In male specimens, the velum makes up 30%–40% of the tail length and the spicula are strongly arcuate (Figure 2h‐j).

***Paralaxus columbae*** sp. nov.

ZooBank registration (http://zoobank.org): urn:lsid:zoobank.org:act:4BABABB3‐1DD4‐46F1‐A69C‐BC06C1271D01.

Species from Florida Keys, USA, can be identified by an amphidial fovea with 1–1.2 turns and the bulbus occupies 29%–33% of the pharynx length. In male specimens, the velum makes up 35%–37% of tail length and the spicula are weakly arcuate (Figure 2k‐o).

### 3.2 *Paralaxus* species from Australia and Hawaii

GenBank entries for 18S rRNA and COI sequences see Table S1.

Representatives of two additional species, *Paralaxus* sp. “heron 1” and *P*. sp. “oahu 1” have been found off Heron Island, Great Barrier Reef, Australia and Oahu, Hawaii Islands. Due to the lack of properly preserved material, a formal description is currently not possible. Morphological details and measurements taken from microphotographs of live animals prior to fixation are presented in the Supplementary Information (Table S2).

Detailed *Paralaxus* species descriptions are presented in the Supplementary Information (Supplementary Results and Figs. S1‐S8).

### 3.3 Key to the species

**Table:** 

Amphidial fovea with 2 turns; bulbus 30%–32% of pharynx length, velum 30%–40% of tail length, spicula strongly arcuate	***Paralaxus bermudensis* sp. nov*.***
Amphidial fovea with 1.5 turns, bulbus 32%–35% of pharynx length, velum 43%–47% of tail length, spicula moderately arcuate	***P. cocos* sp. nov.**
Amphidial fovea with 1–1.2 turns, bulbus 29%–33% of pharynx length, velum 35%–37% of tail length, spicula weakly arcuate	***P. columbae* sp. nov.**

### 3.4 Morphological delineation of the genera *Paralaxus*, *Laxus* and *Leptonemella*

Most characters that identify the genus *Paralaxus* are shared with one or more genera of the Stilbonematinae, but their combination is unique and characteristic. *Paralaxus* is similar to the genus *Laxus* (as reflected in the genus name), but also to the genus *Leptonemella*. The three genera share the extreme forward position of the spiral amphidial fovea, a well‐developed cephalic capsule and a gubernaculum without apophysis. *Paralaxus* can be differentiated from *Laxus* by the lack of a block layer in the cephalic capsule, the presence of a velum on the male tail and in addition by the presence of a multilayered symbiont coat. *Paralaxus* shares the lack of the block layer and the presence of a multilayered symbiont coat with *Leptonemella*, but is distinguished by the greater relative pharynx length b’ (pharynx length/body diameter at end of pharynx), the presence of a velum on the male tail and the lack of sexual dimorphism in the shape of the amphidial fovea.

A special case is *L. brevipharynx* Armenteros, Ruiz‐Abierno, et al. (2014). According to phylogenetic analyses of its 18S and partial COI data (Figure 3a, b), the species belongs to the genus *Leptonemella*. It is morphologically similar to *P. cocos* sp. nov., and despite being molecularly a bona‐fide *Leptonemella,* has a short pharynx and no sexual dimorphism in the shape of the amphidial fovea. There are, however, two morphological features, which clearly separate *L. brevipharynx* from *Paralaxus*: the lack of a velum on the tip of the tail in males and the lack of the long forward directed cephalic setae characteristic for all *Paralaxus* species.

### 3.5 Molecular data corroborates the monophyly of the subfamily Stilbonematinae and all its genera, including *Paralaxus*

In addition to the nine *Paralaxus* specimens, we generated and analysed single worm metagenomes from 20 specimens covering six additional stilbonematine genera and extracted both host 18S and COI gene sequences for all specimens (Table S1). To analyse the monophyly of the Stilbonematinae, we reconstructed their phylogenetic relationships based on the 18S gene, as this is the only gene with sufficient taxon sampling. The 18S gene matrix, with a minimum sequence length of 1,300 bp, consisted of sequences from 78 Stilbonematinae as well as from 52 closely related Desmodorida and Microlaimida, and an outgroup of 24 Chromadorida sequences. The Stilbonematinae formed a highly supported clade, with *other non‐stilbonematine* Desmodoridae and Draconematidae as closest relatives (Figure 3a).

For the analysis of the Stilbonematinae genera, we created a mitochondrial COI tree in addition to the 18S tree, using 27 metagenome‐derived stilbonematine COI sequences and an ascarid as outgroup (*Baylisascaris procyonis* mitochondrion, JF951366) (Figure 3b). Based on both datasets, all Stilbonematinae genera including *Paralaxus* gen. nov. formed well‐supported clades (Figure 3a, b), thus corroborating our morphological assignments. In both the 18S and the COI‐based analysis, the genus *Leptonemella* was phylogenetically most closely related to *Paralaxus*, with high statistical support in the 18S dataset and acceptable support in the COI‐based phylogenies (Figure 3a, b). Other than that, the branching patterns between genera were largely inconsistent between the COI and 18S datasets. This could be linked to the remarkably low support values for many internal nodes in the COI dataset compared to the much higher spectrum of support values for the 18S‐based tree topologies.

In an extended 18S and COI data matrix, we also included eight short‐length, PCR‐amplified sequences of five different Stilbonematinae species with a record of problematic phylogenetic placement—*Laxus parvum, Robbea porosum*, *Stilbonema brevicolle, Catanema exile* and *Leptonemella brevipharynx* (Armenteros, Ruiz‐Abierno, et al., 2014; Figs. S9 and S10). With our new 18S‐ and COI‐based datasets, the placements of *Leptonemella brevipharynx* and *Catanema exile* sequences were resolved and both clustered with sequences of their respective genera. For the sequences of the other three species, we observed inconsistent grouping with Stilbonematinae genera between the datasets (Figs S9 and S10). In the extended 18S tree, two *Stilbonema brevicolle* sequences appeared in two different clades, one closely related to *Leptonemella*, while the other one clustered with a yet undescribed genus‐level clade designated as genus ‘B’ (Zimmermann et al., 2016). In contrast to the 18S‐based placement, the *Stilbonema brevicolle* sequences grouped together in the extended COI tree and formed a novel sister clade to several genera including *Stilbonema*, *Robbea*, *Leptonemella* and *Paralaxus* (Figure S10). In the extended 18S data matrix, the *Laxus parvum* sequences clustered within the genus *Robbea* (Figure S9). Similarly, in the COI dataset the *L. parvum* sequence grouped with the only available sequence of the genus *Robbea* (Figure S10). The sequences designated as *Robbea porosum* formed a divergent genus‐level clade but the placement of this clade was not consistent for the two marker genes (Figs S9 and S10). In the 18S‐based analyses, they were the sister clade to the genus *Laxus*, while in the COI dataset, they formed the sister clade to all other Stilbonematinae.

### 3.6 Symbiont 16S phylogeny shows genus‐level specificity

**Figure 4:**
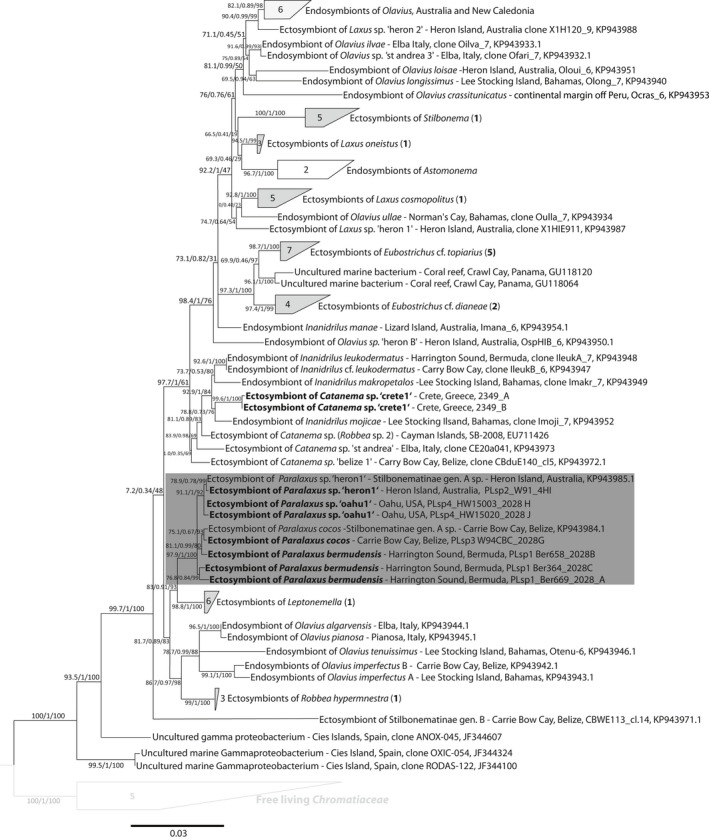
*Candidatus* Thiosymbion phylogeny, including symbionts of *Paralaxus* species and other stilbonematine nematodes, based on the 16S rRNA gene. The tree was calculated using IQTree and node support is given in the following order: SH‐aLRT support (%)/aBayes support/ultrafast bootstrap support (%). Symbionts of species described in this study are marked bold or highlighted in bold numbers next to collapsed branches. Provisional working names for undescribed genera or species are given in quotes. The scale bar represents average nucleotide substitutions per site. Accession numbers for sequences from this study are listed in Table S1

We constructed a matrix from all full‐length 16S ‘Thiosymbion’ sequences, available in the databases and 21 metagenome‐derived symbiont sequences—using five free‐living *Chromatiaceae* as an outgroup. All symbiont sequences from the same host genus clustered in well‐supported clades, irrespective of the number of gutless oligochaete symbionts included within those clades (Figure 4). In congruence to the host data, the symbionts of all newly sequenced *Paralaxus* individuals as well as the two previously published sequences from the Caribbean and Australia (Zimmermann et al., 2016) formed a supported clade in the 16S phylogeny (Figure 4).

### 3.7 COI primers cannot cover the compositional diversity in Stilbonematinae

**Figure 5:**
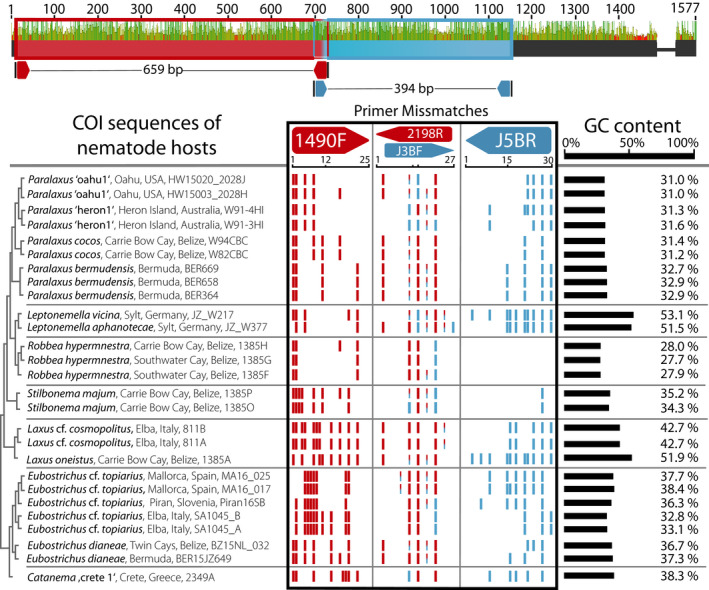
Primer mismatch analysis of the two commonly used COI primer sets. Potential priming sites (forward and reverse) for the primer sets 1490F/2198R (Folmer et al., 1994) and J3BF/J5BR (Armenteros, Rojas‐Corzo, et al., 2014; Armenteros, Ruiz‐Abierno, et al., 2014) along the COI gene are shown in red and blue on the top. The number of mismatches for each primer, when matched to metagenome‐extracted full‐length COI sequences from seven representative stilbonematine nematode genera, is shown below on the left. Average GC content for each full‐length COI sequence is represented by the scale bar on the right

Despite a highly sensitive PCR setup, we could not amplify the COI of any *Paralaxus* specimen using the widely used primer set by Folmer et al. (1994) (data not shown). To elucidate these PCR‐based amplification problems, we analysed all our metagenome‐derived full‐length stilbonematine nematode COI sequences for compositional patterns. The COI sequences of the 19 stilbonematine nematode species that belonged to seven genera had a highly variable guanine and cytosine (GC) content, ranging from 27.7% in the genus Robbea to 53.1% in the genus *Leptonemella*. We identified substantial priming problems with the Folmer et al. (1994) primer set as well with as a second set (JB3F and JB5R) that is commonly used for COI amplification (Armenteros, Rojas‐Corzo, et al., 2014; Armenteros, Ruiz‐Abierno, et al., 2014; Bowles, Blair, & McManus, 1992; Derycke, Vanaverbeke, Rigaux, Backeljau, & Moens, 2010). Across the Stilbonematinae diversity, the Folmer et al. (1994) primers had a range of 3–11 mismatches for the 1490F primer and 1–5 mismatches for the 2198R primer. Similarly, the primers JB3F and JB5R used in a recent study by Armenteros, Ruiz‐Abierno, et al. (2014) showed up to 11 mismatches for each primer (Figure 5). Of all four primers tested, only the JB5R perfectly matched and only to three sequences from *Robbea hypermnestra* (Figure 5). We performed an in silico analysis that mimics PCR conditions of low stringency, which would overcome up to 11 primer mismatches and would cover the whole diversity of Stilbonematinae. Our analysis showed that such permissive conditions would lead to unspecific binding at multiple alternate sites of the COI gene. We tried to design alternative primer sets, but no primer pair, neither for the full‐length nor for partial regions, would reliably target the COI gene and would cover all genera of Stilbonematinae.

### 3.8 The symbiont coat as a new diagnostic feature for Stilbonematinae genera

**Figure 6:**
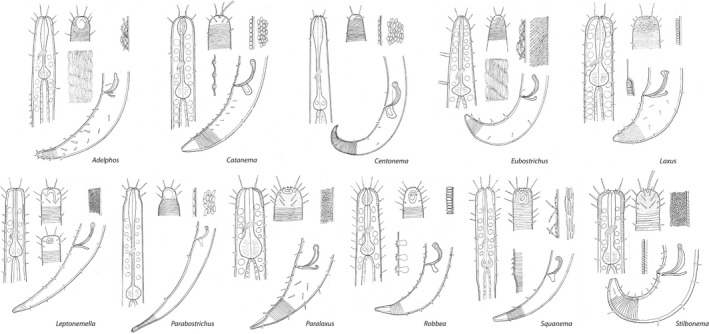
Cellular morphology and coat structure of symbiotic bacteria on different stilbonematine nematode genera. All described genera of Stilbonematinae including *Paralaxus* gen. nov. are depicted in alphabetical order. Each pictogram shows the anterior end of a male in optical section (left), the head region of a male in surface view (top centre), male posterior region with spicular apparatus (lower right) and shape and arrangement of symbionts (upper right). Special features such as arrangement of complex symbiont coat (*Adelphos*, *Eubostrichus*), male supplementary structures (*Catanema*, *Robbea*, *Stilbonema*), sexual dimorphism in the shape of the amphidial fovea (*Leptonemella*) or change in cuticular structure (*Laxus*, *Squanema*) are shown in the centre of the pictogram. Drawings do not represent a defined species but include the general characters of each genus

In addition to several host‐based morphological differences, the presence of a multilayered coat distinguishes the new genus *Paralaxus* from *Laxus*, which carries a monolayer of symbionts. Extending from this observation, we analysed the structure and morphology of the bacterial coat of all Stilbonematinae genera and found substantial and highly informative differences. We therefore propose to include the description of the bacterial coat given below into the genus trait sets as a new character (see also iconographic overview of the different genera in Figure 6).

***Adelphos*** Ott, 1997
Crescent‐shaped bacteria, all cells in contact with host cuticle, double‐attached, forming complex monolayer, covering whole body except anterior and posterior tip.
***Catanema*** Cobb, 1920
Cocci or coccobacilli, all cells in contact with host cuticle, forming simple monolayer, covering whole body except anterior and posterior tip or starting at distance from anterior end.
***Centonema*** Leduc, 2013
Rods (bacilli), lying parallel to cuticle, all cells in contact with host cuticle, forming simple monolayer, probably covering whole body.
***Eubostrichus*** Greeff, 1869
*E. parasitiferus type*
Crescent‐shaped bacteria, all cells in contact with host cuticle, double‐attached, forming complex monolayer, covering whole body except anterior and posterior tip.
*E. dianeae type*
Filamentous bacteria, all cells in contact with host cuticle, singly attached, forming complex monolayer, covering whole body except anterior and posterior tip.
***Laxus*** Cobb, 1894.
Cocci, coccobacilli or rods (bacilli), all cells in contact with host cuticle, rods standing perpendicular to cuticle surface, forming simple monolayer, covering whole body except anterior and posterior tip or starting at distance from anterior end.
***Leptonemella*** Cobb, 1920
Cocci, only a fraction of cells in contact with host cuticle, forming multilayer embedded in gelatinous matrix, covering whole body except anterior and posterior tip.
***Parabostrichus*** Tchesunov et al., 2012
Crescent‐shaped bacteria, all cells in contact with host cuticle, double‐attached, forming complex monolayer, covering whole body except anterior and posterior tip.
***Paralaxus*** gen. nov.
Cocci or coccobacilli, only a fraction of cells in contact with host cuticle, forming multilayer embedded in gelatinous matrix, covering whole body except anterior and posterior tip.
***Robbea*** Gerlach, 1956
Cocci, coccobacilli or rods (bacilli), all cells in contact with host cuticle, rods standing perpendicular to cuticle surface, forming simple monolayer, covering whole body except anterior and posterior tip or starting at distance from anterior end.
***Squanema*** Gerlach, 1963
Crescent‐shaped bacteria, all cells in contact with host cuticle, single‐ or double‐attached, forming monolayer, starting at distance from anterior end.
***Stilbonema*** Cobb, 1920
*S. brevicolle type*
Cocci or rods, all cells in contact with host cuticle, forming simple monolayer covering whole body except for anterior and posterior tip.
*S. majum type*
Cocci, only a fraction of cells in contact with host cuticle, forming multilayer embedded in gelatinous matrix, covering whole body except anterior and posterior tip.

## 4 DISCUSSION

### 4.1 COI‐based barcoding is not suitable for Stilbonematinae

The COI gene is the most common marker gene for PCR‐based animal (meta) barcoding, despite the observation that highly diverse base composition has led to unreliable amplification results in many animal phyla including nematodes (Bhadury et al., 2006; Deagle, Jarman, Coissac, Pompanon, & Taberlet, 2014; Derycke et al., 2010). By utilizing single nematode metagenomics, we could overcome such amplification problems and show that the COI is a valuable phylogenetic marker gene to distinguish stilbonematine genera and species. However, analyses of the metagenome‐derived full‐length COI sequences revealed that within Stilbonematinae the COI gene has a highly diverse base composition, prohibiting the design of specific primers on any region of the COI gene. Our mismatch analyses for commonly used primer sets showed multiple mismatches as well as possible alternate binding sites. One may overcome priming mismatches by using very low stringency PCR conditions, at the cost of specificity. Armenteros, Ruiz‐Abierno, et al. (2014) were able to amplify COI fragments from stilbonematine DNA extracts but morphological species descriptions and 18S‐based results did not conform to the phylogenetic placement based on the COI. One possible explanation could be that the PCR picked up minimal contaminations of other Stilbonematinae with lower mismatch counts, instead of the gene of the target organism with high numbers of mismatches (Figure 5). Together, these factors show that the COI gene is not well suited for PCR‐based barcoding in Stilbonematinae.

### 4.2 Metagenomics‐based molecular data leads to robust phylogenies of Stilbonematinae

Up to this study, 18S and COI genes for Stilbonematinae were PCR‐amplified from DNA extracts, a technique known to be highly susceptible to contamination and bias. This is reflected in two studies by Armenteros, Rojas‐Corzo, et al. (2014) and Armenteros, Ruiz‐Abierno, et al. (2014), which contain phylogenies based on short PCR‐amplified stilbonematine nematode sequences. These studies casted doubt on the monophyly of the subfamily Stilbonematinae, as well as the validity of several stilbonematine genera. As discussed by the authors themselves (Armenteros, Rojas‐Corzo, et al., 2014) and Leduc and Zhao (2016), these conflicting results could have been caused by misidentification of species, PCR bias or artefacts of the phylogenetic reconstruction. To resolve these questions, we used single worm metagenomics from photo‐documented specimens to generate high quality and full‐length 18S and COI sequences that cover the majority of the described stilbonematine genera. Our dataset corroborated the monophyly of the subfamily Stilbonematinae and verified the taxonomic status of all analysed stilbonematine genera. We caution against the use of short and PCR‐based stilbonematine nematode 18S and COI sequences (see Figs. S9 and S10) for phylogenetic analyses and barcoding.

### 4.3 Low coverage next‐generation sequencing should replace PCR‐based barcoding approaches

Instead of using PCR‐based barcoding, a straightforward solution to capture marker genes of a target organism is single individual genome or metagenome sequencing approaches employing next‐generation sequencing (NGS). Our data show that an NGS‐based approach has the advantage that not only single genes but a wide range of marker genes, such as the full rRNA operon as well as the full mitochondrial genome can be recovered from shallow sequencing depths. Recent studies have shown that successful library preparation can be done from as little as 1 picograms of DNA (Rinke et al., 2016) and has been used on a range of microscopic eukaryotic taxa (Gruber‐Vodicka et al., 2019; Jäckle et al., 2019; Seah et al., 2019). Commercially available kits allow input amounts as low as 1 ng of DNA and have been used for low‐cost library construction protocols (Therkildsen & Palumbi, 2017). Low coverage NGS‐based genomic sequencing could even allow for population genetic studies when combined with appropriate replication and probabilistic allele calling techniques (Therkildsen & Palumbi, 2017). Additionally, the associated microbiome is also sequenced, which opens many new avenues for host–microbe research.

### 4.4 The symbiont coat is an additional character of host taxonomy

In microscopic organisms such as marine meiofauna, taxonomic characters are often inconspicuous and not easily recognized. With their relatively simple body plan, only few morphological features are the defining characters for nematode genera. For Stilbonematinae, this leads to the problematic situation that characters overlap and only combinations of multiple characters can delineate genera. This was especially evident in the case of *Paralaxus* gen. nov., as outlined above. The most conspicuous feature of Stilbonematinae is their coat, but initial descriptions of both genera and species often interpreted the obligate symbiotic coat as part of the host (Greeff, 1869), as parasites (Chitwood, 1936) or disregarded the symbiotic coat completely (Cobb, 1920). In his taxonomic review, Tchesunov (2013) gives brief descriptions of their cellular morphologies but does not mention the more informative arrangement of the coat itself.

The fact that *Paralaxus*, but also all other stilbonematine host genera are associated with a specific clade of symbiotic bacteria (based on 16S sequences; Figure 4 and Zimmermann et al., 2016) allows the use of the symbionts’ cellular morphology and coat structure as taxonomic characters for the holobiont. Both cell morphology and coat structure proved to be characteristic for host taxa at the genus level (Figure 6). Interestingly, in the stilbonematine genus *Eubostrichus*, species can host symbionts of either crescent or filamentous shapes (Figure 6). The 18S and COI sequences of hosts harbouring the two morphotypes are phylogenetically distinct (Figure 3a, b), and this clustering is also reflected in the symbiont phylogeny (Figure 4). This suggests that the genus *Eubostrichus* could require a taxonomical reinvestigation. A similar case can be made for the genus *Stilbonema* where at least two coat arrangements (Figure 6) are present but comprehensive molecular data do not exist yet. These two examples emphasize the taxonomic value of the symbiotic coat to reassess the assignment of specimens to genera. Furthermore, coat‐based characters are easily recognized in live specimens as well as in formaldehyde‐preserved material where the coat is often intact. The coat thus facilitates stilbonematine genus‐level differentiation during stereomicroscopic investigations, for example directly in the field. We emphasize that the use of the symbiont coats as additional character will both simplify and enhance Stilbonematinae identification and taxonomy.

## 5 CONCLUSIONS

The last decade has revealed the ubiquitous and intimate link between animals and the microbial world. Conspicuous holobiont features such as the stilbonematine nematode symbiont coats not only support taxonomic identification, but also emphasize the co‐evolution of the symbioses. The highly variable base composition of the Stilbonematinae mitochondrial COI hints at the possible coupling between the obligate and long‐term symbiont and host mitochondrial genomes. These findings also reiterate the importance to analyse more than a single marker gene. We are entering a new age in invertebrate zoology—it is becoming a viable option to employ data‐intensive NGS sequencing for multilocus analyses and, when performed in a shotgun metagenomic approach, this allows to consider the permanently associated microbiome at the same time. Exploring these options, our results show how metagenomics and the integration of holobiont features can shape taxonomic studies and likely many more fields of zoology in the future.

## Data Availability

Sequences from this study were submitted to the European Nucleotide Archive (ENA) using the data brokerage service of the German Federation for Biological Data GFBio (Diepenbroek et al., 2014; https://www.gfbio.org) and are available under the study accession number PRJEB27096 ‐ 16S: LR746242‐LR746262;18S: LR746196‐LR746218;COI: LR746312‐LR746338.
