# Supplementary material for: Morphology of obligate ectosymbionts reveals Paralaxus gen. nov.: A new circumtropical genus of marine stilbonematine nematodes
Source: Zool Scr. 2020 Feb 20;49(3):379–94. doi: 10.1111/zsc.12399 (PMC8614112; doi:10.1111/zsc.12399)
Supplement: Supplementary file 1 [file ZSC-49-379-s001.pdf]

Supplementary Information to

Morphology of obligate ectosymbionts reveals *Paralaxus* gen. nov., a new circumtropical genus of marine stilbonematine nematodes

Florian Scharhauser[1*], Judith Zimmermann[2*], Jörg A. Ott[1], Nikolaus Leisch[2] and Harald R. Gruber-Vodicka[2]

[1]Department of Limnology and Bio-Oceanography, University of Vienna, Althanstrasse 14, A-1090 Vienna, Austria

[2] Max Planck Institute for Marine Microbiology, Celsiusstrasse 1, D-28359 Bremen, Germany

SI Results

Descriptions

Class Chromadorea Inglis, 1983

Subclass Chromadoria Pearse, 1942

Order Desmodorida De Coninck, 1965

Suborder Desmodorina De Coninck, 1965

Superfamily Desmodoroidea Filipjev, 1922

Family Desmodoridae Filipjev, 1922

Subfamily Stilbonematinae Chitwood, 1936

***Paralaxus* gen. nov.**

http://zoobank.org: 417691DB-5EE3-49B7-B838-1BBF1569235F

Diagnosis. Stilbonematinae. Cuticle transversely striated, except for the head region and the tip of the tail. The striation is coarser in the anterior region and transitions to a finer striation after three to four pharynx lengths. Cephalic capsule distinct, without a block-layer, finely punctated; cephalic setae as long or longer than the sub-cephalic setae, usually directed straight forward. Amphidial fovea an open spiral, ventrally wound, in extreme forward position. No sexual dimorphism in the shape of the amphidial fovea. Pharynx bulbus large, muscular. Gubernaculum dorsally directed with terminal hook, without apophysis. Males have a distinct velum at the tip of the tail. Glandular sense organs (gso) enlarged ventrally in post-pharyngeal region in males; anterior and posterior to vulva in females. Body covered by a multi-layered coat of symbiotic coccobacilli.

Etymology: Superficially resembling species of the genus *Laxus* Cobb.

Type species: *Paralaxus cocos* sp. nov.

Symbiont Diagnosis. The bacterial coat consists of coccobacilli shaped cells between 0.8 µm to 1.7 µm in size, which are distributed over the whole body, leaving only the more coarsely annulated anterior region and the tip of the tail uncovered. They are

arranged in a thick multilayer from 6 up to 12 layers of bacteria, which are embedded in a gelatinous matrix showing no layer separation or structure. Only bacterial cells at the bottom of the coat are in contact with the cuticle of the nematode host. Most cells show pili-like structures that are connected to the worm surface and/or to neighbouring bacteria. The bacterial cells divide transversely.

Paralaxus cocos sp. nov.
(Figs. 2 – 23)
ZooBank registration: http://zoobank.org A659918E-FD88-4584-8115-CD4C396AE400
Type material: Holotype (male), 3 paratypes (male), 3 paratypes (female), 4 paratypes (juvenile), deposited at the Natural History Museum Vienna (accession numbers NHMWZooEvMikro 5689 - 5699)

Measurements. See Table S2

Additional material: Several specimens in the collection of JAO and those used for SEM.

Type locality: Subtidal fine sand among Red Mangrove (*Rhizophora mangle*) roots and turtle grass (*Thalassia testudinum*), 10 – 50 cm depth, W-side of Twin Cayes, BELIZE (16°49'45.6"N 88°06'28.9"W)

Distribution: Regularly in fine to medium subtidal sands around Carrie Bow Cay, Curlew Cay and Twin Cayes (Belize).

Etymology: From the Latin name of the Coconut Tree, *Cocos nucifera*, one of which played an important role in the research trip that led to the discovery of the new genus.

Description:

Body cylindrical, tail conical.

Cuticle transversely striated except for the first 25 - 30 µm of the head and the last 40 - 50 µm of the tail (Fig. 14), striae 0.6 to 0.7 µm wide (14 - 16 striae/10 µm) in anterior body part, 0.4 -0.5 µm (20 - 23 striae/10µm) after 3 – 3.7 pharynx length from anterior end. The anterior most circle of head sensillae (inner labial sensillae) is represented by 6 spoon-shaped papillae within the mouth opening, in lateral, subventral and subdorsal position. The second circle consists of 6 short outer labial sensillae on the margin of the membranous buccal field in lateral, subventral and subdorsal position; 4 cephalic setae near the anterior margin of the amphidial fovea; 3 circles of 8 subcephalic setae each, on the non-striated part of the head region, the submedian rows with additional short setae, 10 µm long; 8 rows of somatic setae along the whole length of the body, spaced 20 µm apart. Non-striated part of the tail in females with 2 pairs of setae, in males with a velum. Three caudal glands extending to the gubernaculum. Amphidial foveas situated at the anterior end bordering the buccal field, an open spiral, ventrally wound, with 1.5 turns.

Pharynx with a minute buccal cavity, leading into a slightly dilated corpus, which is only indistinctly separated from the isthmus. Posterior bulbus spherical, muscular, large. No cardia. The bulbus to pharynx ratio is 32 – 35% in males and 30 - 34% in females.

Nerve ring 45 - 55 µm from anterior end; no secretory-excretory pore or ventral gland seen; 8 rows of glandular sense organs (two in each lateral and each median line).

Males monorchic, testis on the right side of the intestine; spicula strong, arcuate, cephalate proximally; gubernaculum straight, corpus embracing spicules, end hook shaped. Sperm globular. Row of enlarged gso along the mid-ventral line 400 µm long, beginning at 250 µm from the anterior end.

Females didelphic, ovaries reflexed. Vulva with anterior and posterior vulvar gland, no special sclerotization. Enlarged ventral gso anterior and posterior to vulva, extending 250 µm in either direction.

Paralaxus bermudensis sp. nov.

(Figs. 24 – 30)

ZooBank registration: http://zoobank.org 8697C6A0-3508-4833-81DC-D2A86B0B00AC

Type material: Holotype (male), 1 paratype (male), 2 paratypes (juvenile), deposited at the Natural History Museum Vienna (accession numbers NHMWZooEvMikro 5700 - 5703)

Measurements. See Table S2

Type locality: Subtidal sand, 3 m depth, NW of Nonsuch Island, BERMUDA (32°20' 54'' N, 64° 39' 54'' W)

Distribution: Also found in subtidal sand in Harrington Sound and Baylis Bay, Bermuda.

Etymology: From the Bermuda Islands

Description:

Body cylindrical, tail conical.

Cuticle transversely striated except for the first 30 - 35 µm of the head and the last 40 - 45 µm of the tail, striae 1 – 1.1 µm wide (9 - 10 striae/ 10 µm) in anterior body part, appr. 0.5 µm (20/ 10 µm) after 3.2 – 3.4 pharynx lengths from anterior end. The anterior most circle of head sensillae (inner labial sensillae) not seen, the second circle as in *P. cocos*; 4 cephalic setae near the anterior margin of the amphidial fovea; 3 circles of 8 subcephalic setae each, with additional short setae, 10 µm long on the non-striated part of the head region; 8 rows of somatic setae along the whole length of the body, spaced 20 µm apart. Tip of tail in males with a velum. Three caudal glands extending to the gubernaculums. Amphidial foveas with 2 turns.

Pharynx with a minute buccal cavity, corpus slightly dilated. Bulbus spherical, muscular, large, no cardia. The bulbus to pharynx ratio is 31 – 33%.

Nerve ring 55 – 65 µm from anterior end; no secretory-excretory pore or ventral gland seen; 8 rows of glandular sense organs (two in each lateral and each median line).

Males monorchic, testis on the right side of the intestine; spicula strongly arcuate, cephalate proximally; gubernaculum straight, end hook shaped. Row of enlarged GSO beginning at end of pharynx and extending 250 µm backwards.

No female found.

Paralaxus columbae sp. nov.

(Figs. 31 – 39)

ZooBank registration: http://zoobank.org 4BABABB3-1DD4-46F1-A69C-BC06C1271D01

Type material: Holotype (male), 1 paratype (male), 3 paratypes (female), 2 paratypes (juvenile), deposited at the Natural History Museum Vienna (accession numbers NHMWZooEvMikro 5704 - 5710)

Measurements. See Table S2

Additional material: Several specimens in the collection of JAO.

Type locality: Subtidal sand, 2 m depth, N-side of Pigeon Key, Florida, USA (24° 42' 19'' N, 81° 09' 20'' W)

Distribution: Only known from type locality.

Etymology: From the Latin *columba* (pigeon), referring to the type locality.

Description:

Body cylindrical, tail conical.

Cuticle transversely striated except for the first 30 - 35 µm of the head and the last 40 - 50 µm of the tail, striae 0.6 to 0.7 µm wide (14 - 16 striae/10 µm) in anterior body part, 0.4 -0.5 µm (20 - 23 striae/10µm) after 3 – 3.2 pharynx length from anterior end.

The anterior most circle of head sensillae (inner labial sensillae) not seen. The second circle as in *P. cocos*; 4 cephalic setae at the level of the amphidial fovea; 3 circles of 8 subcephalic setae each, on the non-striated part of the head region, the submedian rows with additional short setae, 10 µm long; 8 rows of somatic setae along the whole length of the body, spaced 15 - 20 µm apart. Males with a velum. Three caudal glands extending to the gubernaculum. Amphidial foveas situated at the anterior end, an open spiral with 1 – 1.2 turns, ventrally wound.

Pharynx with minute, buccal cavity, corpus not dilated. Posterior bulbus subspherical, muscular, large. No cardia. The bulbus to pharynx ratio is 29 - 33% in both sexes. Nerve ring 45 - 55 µm from anterior end; no secretory-excretory pore or ventral gland seen; 8 rows of glandular sense organs (two in each lateral and each median line).

Males monorchic, testis on the right side of the intestine; spicula weakly arcuate, cephalate proximally; gubernaculum straight, corpus embracing spicules, end hook shaped. Enlarged GSO along the mid-ventral line beginning at end of the pharynx and extending 300 - 350 µm backwards.

Females didelphic, ovaries reflexed. Vulva as in *P. cocos* sp. nov. Enlarged ventral gso anterior and posterior to vulva extending approximately 200 µm in either direction.

Paralaxus species from Australia and Hawaii

Two additional *Paralaxus* species, one from Australia and one from Hawaii have been discovered by sequencing their 18S rRNA and COI genes alone. Due to the lack of properly preserved material, a formal description is therefore currently not possible. However, we were able to make measurements and morphological descriptions from microphotographs that were taken from the live animals prior to sequencing, which are listed below:

***Paralaxus* sp. ``heron 1´´**

(Figs. 40 – 42)

Sample location: Heron Island, Great Barrier Reef, AUSTRALIA (24° 26’ 36’’ S, 151°54’ 47’’ W)

Material: 1 female

Description (measurements taken from LM micrographs of sequenced specimen)

Since the total length could not be obtained from the micrographs, the parameters a, b and c could not be calculated. Maximum diameter at the level of a fully developed egg was 86 µm, in other regions of the body 78 µm, which was also the diameter at the end of the pharynx. Cuticle striated, striae 1.2 to 1.3 µm wide. Pharynx 122 µm long, bulbus 41 µm long (33.6% of pharynx length) and 45 µm wide. Cephalic capsule 28 µm long, bacterial coat thick, consisting of coccobacilli approximately 1 µm long.

***Paralaxus* sp. ``oahu 1´´**

(Figs. 43 – 45)

Sample locations: Oahu, Hawaii Islands, USA (21° 28’ 11,3’’ N, 157° 49’ 4,8’’ W and 21° 16’ 50,4’’ N, 157° 43’ 40,8 W)

Material: 2 individuals, at least 1 male.

Description (measurements taken from LM micrographs of sequenced specimens)

Length 4927 µm

a= 71.4; b= 37.3; c= 35.7

Length of pharynx 132 µm, length of tail 138 µm; maximum diameter 69 µm, head diameter 41 µm, diameter at end of pharynx 58 µm, cloacal diameter 62 µm, c’= 2.2. Cuticle transversely striated, except for the cephalic capsule (34 µm long) and the tip of the tail, striae 1 µm wide, head and body setation not discernible in micrographs. Amphids in extreme forward position, open spiral with one turn, 13.5 µm wide, 9 µm

long. Pharynx bulbus 36.4 μm wide and 38.8 μm long (29.6% of pharynx length). Spicula cephalate, curved, 64 μm (arc), 58 μm (chord) long, gubernaculum straight with terminal hook, 34.5 μm long. Several layers of coccobacilli approximately 1 μm long covering body.

SI Tables

Table S1: Stilbonematine nematode specimens sampled for this study

Sample ID / Library	Host species	Sampling region	Ocean region	Location (Long/Lat)	Sampling date	Habitat (marine zone)	Habitat (sediment grain size)	Climate	Accession no. host 18S rRNA gene	Accession no. host COI gene	Accession no. symbiont 16S gene
W91-3HI / 2028D	*Paralaxus* "heron 1"	Heron Island, Australia	SW Pacific, Coral Sea	-23.44339, 151.91307	Aug, 2012	intertidal	coarse	tropical	KP943969 (Zimmermann et al., 2016)	LR746325	KP943985 (Zimmermann et al., 2016)
W91-4HI / 2028E	*Paralaxus* "heron 1"	Heron Island, Australia	SW Pacific, Coral Sea	-23.44339, 151.91307	Aug, 2012	intertidal	coarse	tropical	LR746207	LR746326	**R746251**
W94CBC / 2028G	*Paralaxus cocos*	Twin Cays, Belize	W Atlantic, Caribbean Sea	16.82861, -88.10955	Apr, 2013	subtidal	very fine	tropical	LR746206	LR746333	LR746254
W82CBC / 2028F	*Paralaxus cocos*	Twin Cays, Belize	W Atlantic, Caribbean Sea	16.82861, -88.10955	Apr, 2013	subtidal	very fine	tropical	KP943968 (Zimmermann et al., 2016)	LR746332	KP943984 (Zimmermann et al., 2016)

Sample ID / Library	Host species	Sampling region	Ocean region	Location (Long/Lat)	Sampling date	Habitat (marine zone)	Habitat (sediment grain size)	Climate	Accession no. host 18S rRNA gene	Accession no. host COI gene	Accession no. symbiont 16S gene
BER669 / 2028A	*Paralaxus bermudensis*	Harrington Sound, Bermuda	N Atlantic	32.324038, -64.738267	May, 2015	subtidal	medium coarse	subtropical	LR746205	LR746331	LR746255
BER658 / 2028B	*Paralaxus bermudensis*	Harrington Sound, Bermuda	N Atlantic	32.324038, -64.738267	May, 2015	subtidal	medium coarse	subtropical	LR746204	LR746330	LR746250
BER364 / 2028C	*Paralaxus bermudensis*	Harrington Sound, Bermuda	N Atlantic	32.324038, -64.738267	May, 2015	subtidal	medium coarse	subtropical	R746203	LR746329	LR746249
JO93-1 / -	*Paralaxus bermudensis*	Bermuda, Nonsuch Island	N Atlantic	32.3477777, -64.665	1993	subtidal	medium fine	subtropical	-	-	-
HW15020 / 2028J	*Paralaxus* "oahu 1"	Oahu, Hawaii	Central Pacific	21.280897, -157.728126	Oct, 2015	intertidal		tropical	LR746209	LR746328	LR746253

Sample ID / Library	Host species	Sampling region	Ocean region	Location (Long/Lat)	Sampling date	Habitat (marine zone)	Habitat (sediment grain size)	Climate	Accession no. host 18S rRNA gene	Accession no. host COI gene	Accession no. symbiont 16S gene
HW15003 / 2028H	*Paralaxus* "oahu 1"	Oahu, Hawaii	Central Pacific	21.280897, -157.728126	Oct, 2015	intertidal		tropical	LR746208	LR746327	LR746252
JO70-1 / -	*Paralaxus columbae*	Florida	Atlantic	24.705277777, -81.1555555	May, 1970	subtidal	medium fine	subtropical	–	–	–
HGV13CBC-01 / 1385H	*Robbea hypermnestra*	Carrie Bow Cay, Belize	W Atlantic, Caribbean Sea	16.81311, -88.08299	Apr, 2013	subtidal	coarse	tropical	no sequence	LR746336	no sequence
HGV13CBC-02 / 1385F	*Robbea hypermnestra*	Southwater Cay, Belize	W Atlantic, Caribbean Sea	16.81311, -88.08299	Apr, 2013	subtidal	coarse	tropical	no sequence	LR746334	no sequence
HGV13CBC-03 / 1385G	*Robbea hypermnestra*	Southwater Cay, Belize	W Atlantic, Caribbean Sea	16.81311, -88.08299	Apr, 2013	subtidal	coarse	tropical	LR746216	LR746335	LR746256

Sample ID / Library	Host species	Sampling region	Ocean region	Location (Long/Lat)	Sampling date	Habitat (marine zone)	Habitat (sediment grain size)	Climate	Accession no. host 18S rRNA gene	Accession no. host COI gene	Accession no. symbiont 16S gene
JZ_W217 / 1094A	*Leptonemella vicina*	List, Sylt	North Sea	55.01467, 8.43752	June, 2013	intertidal	fine	temperate	KU921488	LR746324	KU921521
JZ_W377/ 1855C	*Leptonemella aphanothecae*	List, Sylt	North Sea	55.01467, 8.43752	Sept, 2015	intertidal	fine	temperate	LR746210	LR746323	LR746248
NL14CBC-01 / 1385P	*Stilbonema majum*	Carrie Bow Cay, Belize	W Atlantic, Caribbean Sea	16.8030556, -088.0819444	Apr, 2014	subtidal	coarse	tropical	LR746214	LR746338	no sequence
NL14CBC-02 / 1385O	*Stilbonema majum*	Carrie Bow Cay, Belize	W Atlantic, Caribbean Sea	16.8030556, -088.0819444	Apr, 2014	subtidal	coarse	tropical	LR746213	LR746337	no sequence
NL14CBC-03 / 2348E	*Stilbonema majum*	Carrie Bow Cay, Belize	W Atlantic, Caribbean Sea	16.8030556, -088.0819444	Apr, 2013	subtidal	coarse	tropical	LR746215	no sequence	LR746260

**Sample ID / Library**	**Host species**	**Sampling region**	**Ocean region**	**Location (Long/Lat)**	**Sampling date**	**Habitat (marine zone)**	**Habitat (sediment grain size)**	**Climate**	**Accession no. host 18S rRNA gene**	**Accession no. host COI gene**	**Accession no. symbiont 16S gene**
NL14CBC-04 / 1385A	*Laxus oneistus*	Carrie Bow Cay, Belize	W Atlantic, Caribbean Sea	16.8030556, -088.0819444	March, 2014	subtidal	coarse	tropical	LR746201	LR746322	LR746261
HGVE13-03 / 811Aand 703A	*Laxus* cf. *cosmopolitus*	Elba, Italy	Mediterranean Sea	42.808366, 10.141731	Aug, 2013	subtidal	medium coarse	temperate	LR746202	LR746320	LR746259
HGVE13-04 / 811B	*Laxus* cf. *cosmopolitus*	Elba, Italy	Mediterranean Sea	42.808366, 10.141731	Aug, 2013	subtidal	medium coarse	temperate	no sequence	LR746321	no sequence
NL15CR-01 / 2349A	*Catanema* "crete 1"	Crete, Greece	Mediterranean Sea	35.515099, 23.989591	Aug, 2015	subtidal	medium fine	temperate	R746211	LR746312	LR746257
NL15CR-02 / 2349B	*Catanema* "crete 1"	Crete, Greece	Mediterranean Sea	35.515099, 23.989591	Aug, 2015	subtidal	medium fine	temperate	LR746212	no sequence	LR746258

**Sample ID / Library**	**Host species**	**Sampling region**	**Ocean region**	**Location (Long/Lat)**	**Sampling date**	**Habitat (marine zone)**	**Habitat (sediment grain size)**	**Climate**	**Accession no. host 18S rRNA gene**	**Accession no. host COI gene**	**Accession no. symbiont 16S gene**
MA16_017 / 2344_L	*Eubostrichus* cf. *topiarius*	Mallorca, Spain	Mediterranean Sea	39.516388, 2.741944	May, 2016	subtidal	fine	temperate	**LR746198**	**LR746313**	**R746246**
MA16_025 / 2344_J	*Eubostrichus* cf. *topiarius*	Mallorca, Spain	Mediterranean Sea	39.757055, 3.219944	May, 2017	subtidal	fine	temperate	**LR746199**	**LR746314**	**LR746244**
Piran16SB_N6 / 2349_L	*Eubostrichus* cf. *topiarius*	Piran, Slovenia	Mediterranean Sea	45.517656, 13.568039	August, 2016	subtidal	fine	temperate	**LR746197**	**LR746315**	**LR746245**
HGVE13-01 / 1045A	*Eubostrichus* cf. *topiarius*	Elba, Italy	Mediterranean Sea	42.808366, 10.141731	Aug, 2013	subtidal	medium coarse	temperate	**LR746196**	**LR746316**	**LR746242**
HGVE13-02 / 1045B	*Eubostrichus* cf. *topiarius*	Elba, Italy	Mediterranean Sea	42.808366, 10.141731	Aug, 2013	subtidal	medium coarse	temperate	**LR746200**	**LR746317**	**LR746243**

Sample ID / Library	Host species	Sampling region	Ocean region	Location (Long/Lat)	Sampling date	Habitat (marine zone)	Habitat (sediment grain size)	Climate	Accession no. host 18S rRNA gene	Accession no. host COI gene	Accession no. symbiont 16S gene
BZ15NL_032 / 2348L	*Eubostrichus dianeae*	Twin Cays, Belize	W Atlantic, Caribbean Sea	16.823702, -88.106056	May, 2015	subtidal	fine	tropical	**LR746218**	**LR746319**	**LR746262**
BER15JZ649 / 2349H	*Eubostrichus dianeae*	Harrington Sound, Bermuda	N Atlantic	32.324038, -64.738267	May, 2015	subtidal	medium coarse	subtropical	**LR746217**	**LR746318**	**LR746247**

Table S2. Morphological details and measurements [all given in µm] taken from microphotographs of live specimens of *Paralaxus cocos* sp. nov., *P. bermudensis* sp. nov. and *P. columbae* sp. nov. (n – number of analysed individuals, a b c - demanian proportions, n.d. – not determined, n.a. – not applicable)

	Paralaxus cocos **sp.nov.**			***Paralaxus bermudensis*** **sp.nov.**		***Paralaxus columbae*** **sp.nov.**		
	Holotype	Paratype male	Paratype female	Holotype	Paratype male	Holotype	Paratype male	Paratype female
n	1	3	3	1	1	1	1	3
Length	3822	3135 -4602	4336 - 5069	4115	4336	3568	2801	3935 - 4669
a	73.5	56.9 - 83.6	72.3 - 94.3	68.6	66.7	64.9	50.9	66 - 78
b	42.9	28.5 - 43	36.7 - 43.4	35.5	34.7	32.4	28	35.8 - 44.5
c	31.8	27.3 - 41.7	40.8 - 42.9	39.2	34.7	31.8	25.9	36.4 - 39.7
max. diameter	52	50 - 55	46 - 66	60	65	55	55	55 - 60
pharynx length	89	98 - 110	100 - 130	116	125	110	100	103 - 110
tail length	120	108 - 115	102 - 118	105	125	112	108	100 – 120

	Paralaxus cocos sp.nov.			*Paralaxus bermudensis* sp.nov.		*Paralaxus columbae* sp.nov.		
	Holotype	Paratype male	Paratype female	Holotype	Paratype male	Holotype	Paratype male	Paratype female
head diameter	20	21 - 25	16 - 20	35	25	33	40	32 - 35
amphid c.b.d.	28	23 - 28	24 - 25	45	50	40	42	45 - 50
anal/cloacal diameter	50	50 - 55	40 - 42	60	65	55	52	50 - 55
c' (tail length/anal dia.)	2.4	2.0 - 2.3	2.6 - 2.8	1.75	1.9	2	1.96	1.8 - 2.2
outer lab. sensillae length	1	1	1	1	1	1	1	1
cephalic setae length	22	20 - 28	21 - 25	25	20	30	25	25 - 28
subcephalic setae length	16	15 - 17	18 - 20	12 - 20	13 - 15	15 - 20	15 - 19	15 - 17
somatic setae length	12	9 - 12	10 - 13	8 - 9	9 - 10	8	7 – 10	9 - 10
testis begin %	36	36 - 37	n.a.	40	n.d.	45	45	n.a.
vulva %	n.a.	n.a.	44 - 45	n.a.	n.a.	n.a.	n.a.	44.5 - 48
amphidial fovea width	14	14 - 15	11 - 12	15	12	12	12	12 - 13

	Paralaxus cocos **sp.nov.**			***Paralaxus bermudensis*** **sp.nov.**		***Paralaxus columbae*** **sp.nov.**		
	Holotype	Paratype male	Paratype female	Holotype	Paratype male	Holotype	Paratype male	Paratype female
amphidial fovea length	10	10 - 15	6 - 8	10	12	12	12	12 - 13
pharynx bulbus length	30	32-37	30-45	37	42	35	30	30-35
pharynx bulbus width	28	32-35	27-43	35	40	32	35	37-45
bulbus % of pharynx l	33.7	31.8 - 34.5	30 - 34.6	31.9	32	31.8	30	29 - 33
diam. end of pharynx	47	46 - 57	46 - 50	60	65	55	54	55 - 60
spicula length arc	61	60-65	n.a.	57	63	60	60	n.a.
spicula length cord	50	50-51	n.a.	45	45	50	50	n.a.
gubernaculum length	33	31 - 35	n.a.	30	30	30	32	n.a.
velum % of tail length	47	43 - 46	n.a.	40	36	35.7	37	n.a.

235 SI Figures

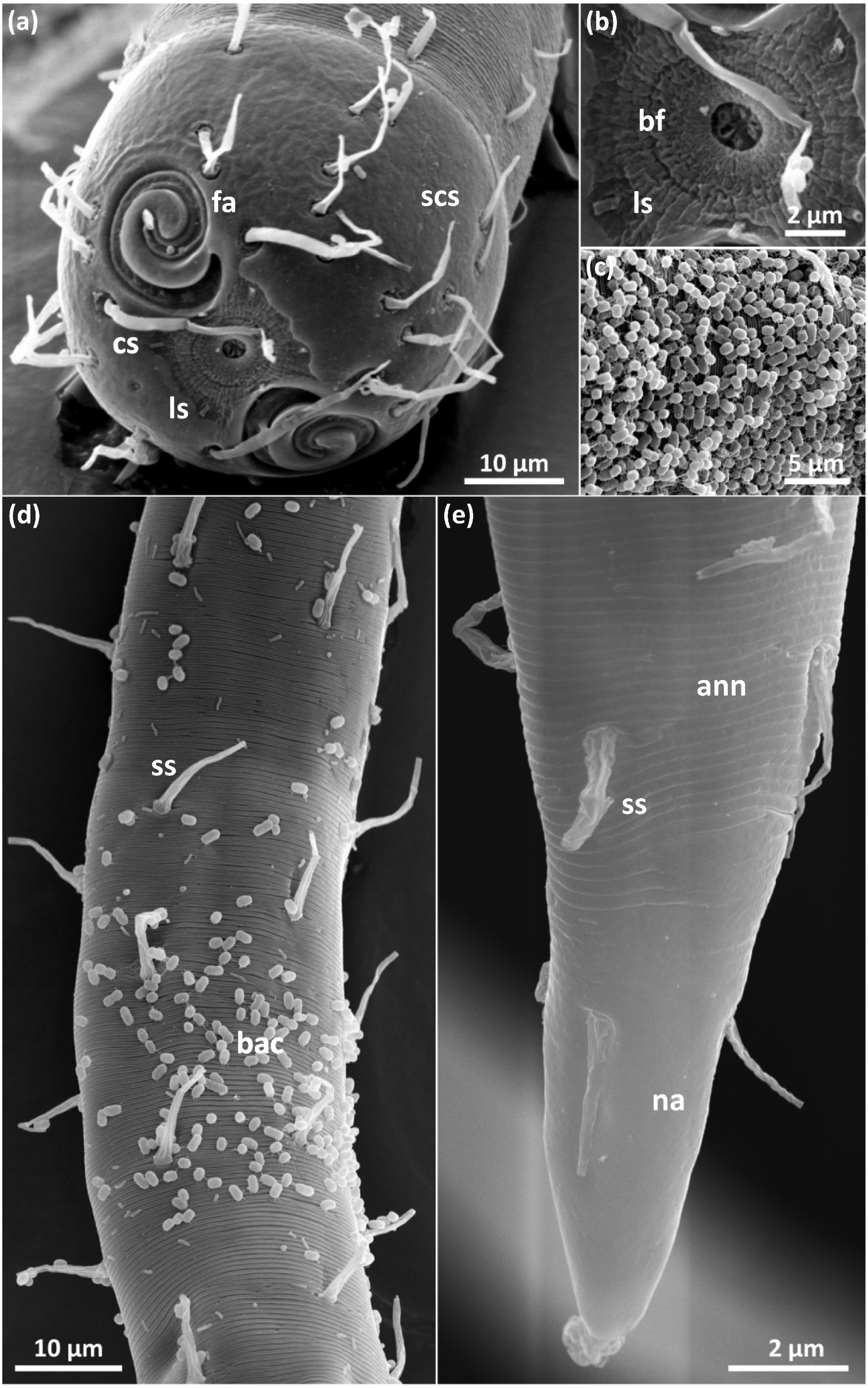

Fig. S1. Scanning electron micrographs of *Paralaxus cocos* sp. nov. female (a) Anterior in *en face* view, (b) buccal field, (c) detail of symbiotic coat, (d) midbody region, (e) tip of tail. SEM (ann annulation, bac symbiotic bacteria, bf buccal field, cs cephalic setae, fa fovea amphidalis, ls labial setae, na non-annulated tip of tail, scs short-cephalic setae, ss somatic setae.)

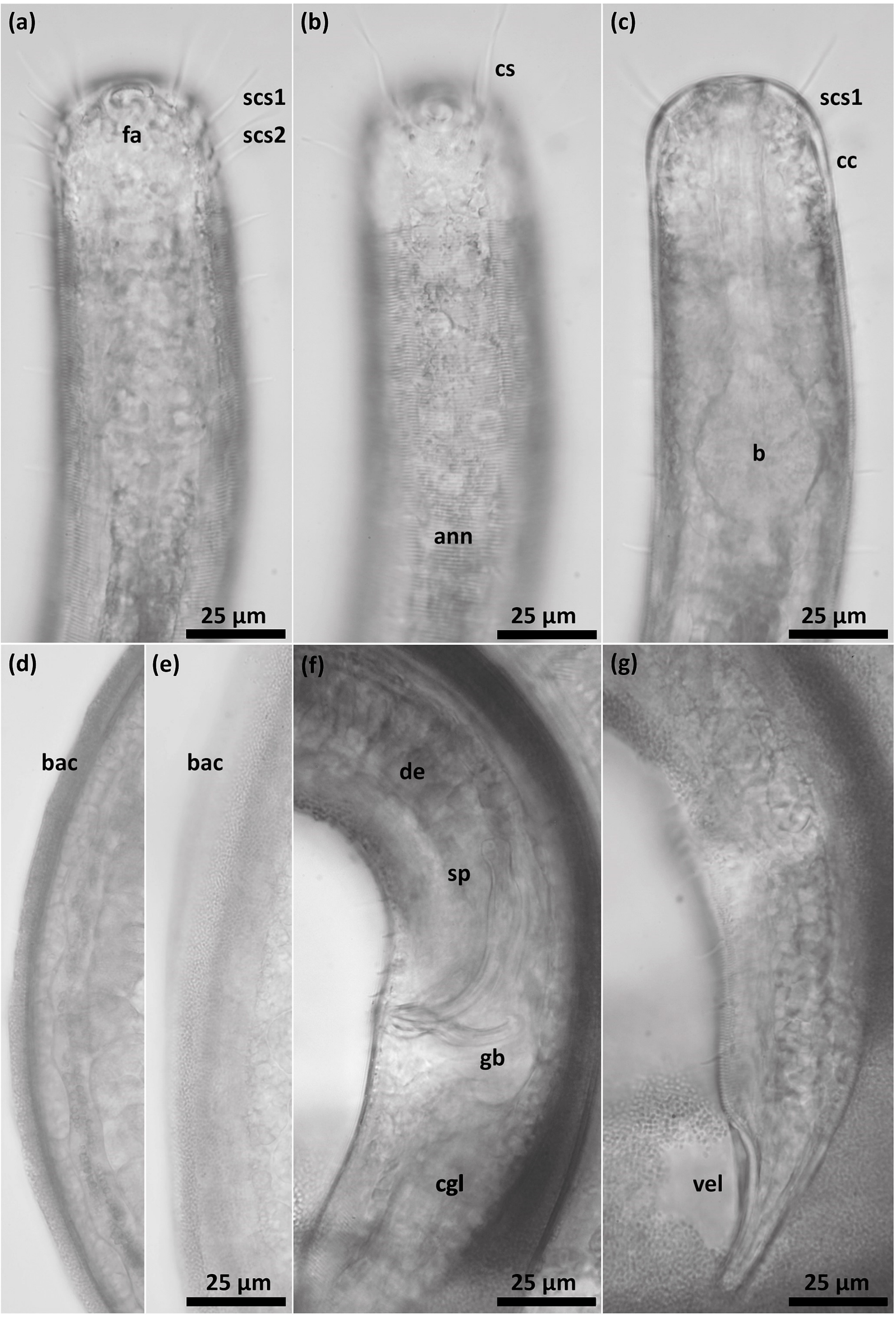

Fig. S2. Light micrographs of live specimen of *Paralaxus cocos* sp. nov. male. (a) anterior end (b) anterior end surface view (c) anterior end, optical section (d) midbody region, optical

section of bacterial coat (e) midbody region, surface view of bacterial coat (f) cloacal region (g) tip of tail (ann annulation, b pharyngeal bulbus, bac symbiotic bacteria, cc cephalic capsule, cgl caudal gland, cs cephalic setae, de ductus ejaculatorius, fa fovea amphidalis, gb gubernaculum, scs1 subcephalic setae of first circle, scs2 subcephalic setae of second circle, sp spiculum, vel velum).

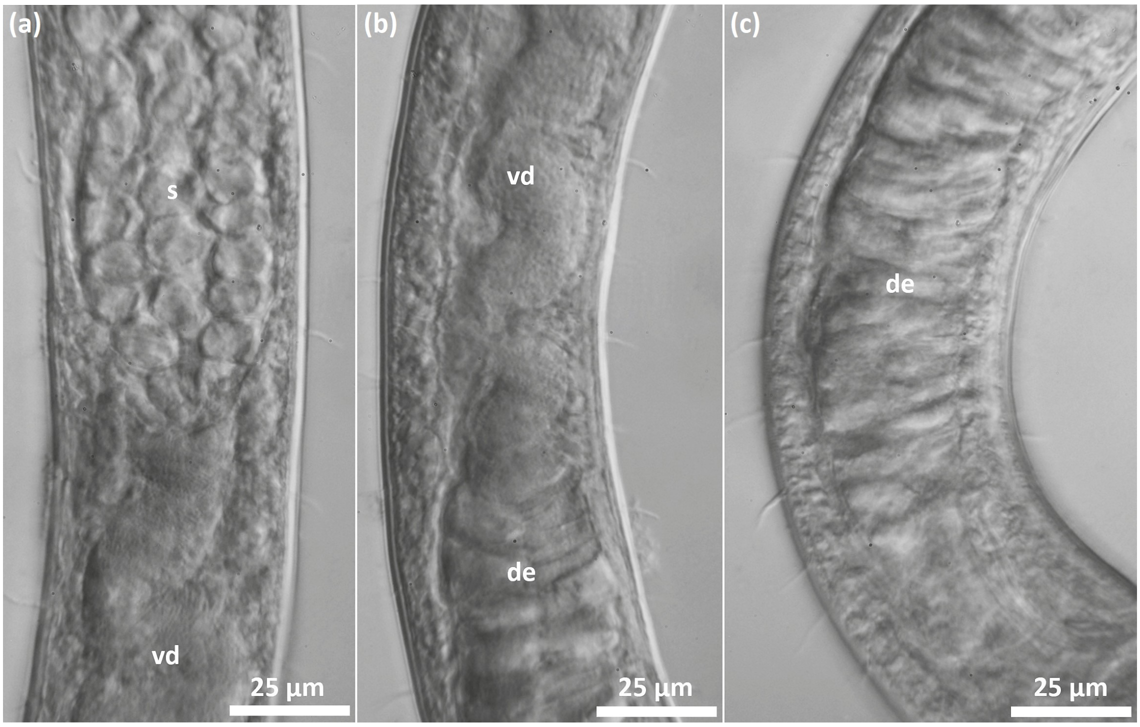

Fig. S3. Light micrographs of the reproductive system of *Paralaxus cocos* sp. nov. male. (a) junction of testis and vas deferens (b) junction of vas deferens and ductus ejaculatorius (c) posterior end of ductus ejaculatorius. LM of live specimen. (de ductus ejaculatorius, s sperm, vd vas deferens)

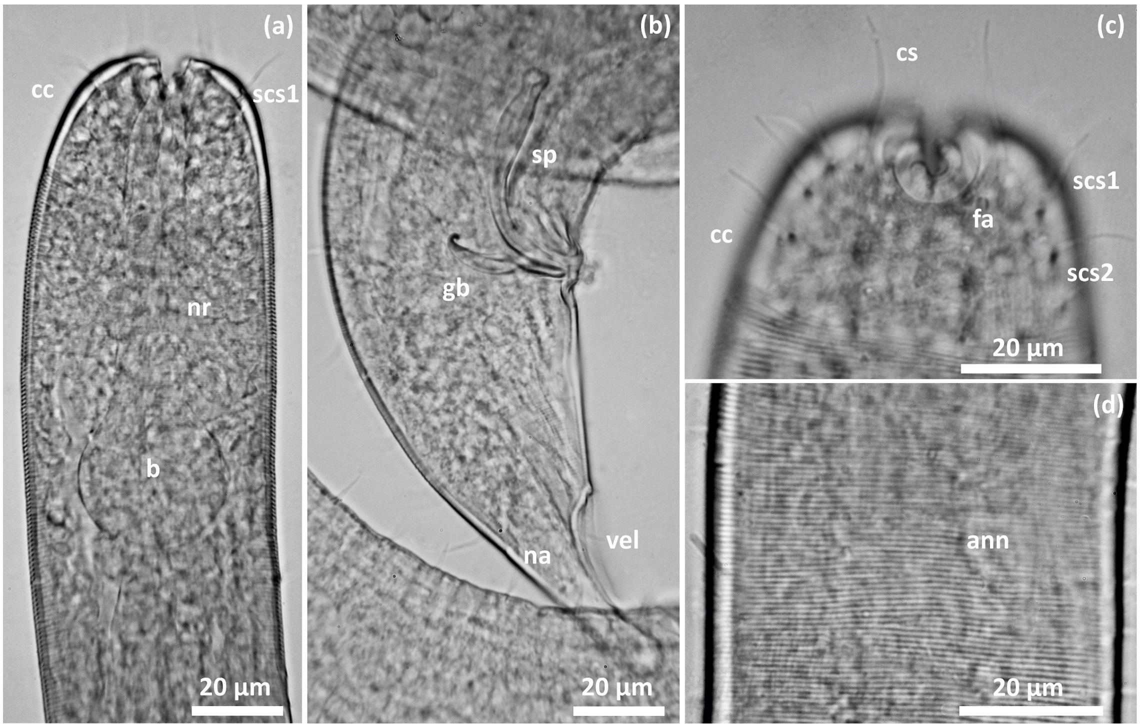

Fig. S4. Light micrographs of preserved specimens of *Paralaxus bermudensis* sp. nov. male. (a) anterior end, optical section (b) posterior end (c) anterior tip, surface view (d) midbody region (ann annulation, b pharyngeal bulbus, cc cephalic capsule, cs cephalic setae, fa fovea amphidalis, gb gubernaculum, na non-annulated tip of tail, nr nerve ring, scs1 subcephalic setae of first circle, scs2 subcephalic setae of second circle, sp spiculum, vel velum).

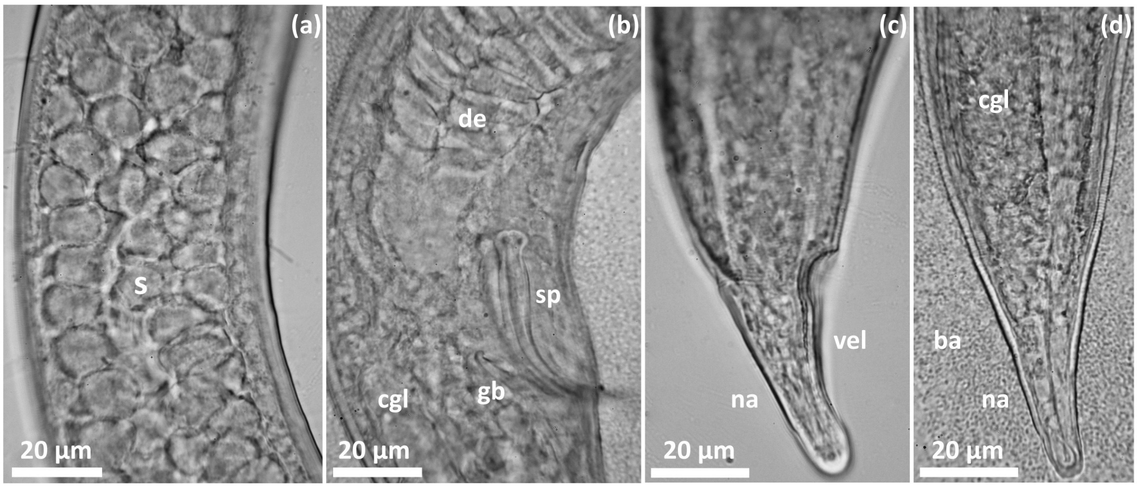

Fig. S5. Light micrographs of the posterior end of preserved specimen of *Paralaxus columbae* sp. nov. (a – c) male (a) sperm in testis (b) cloacal region (c) tail (d) female tail (ba symbiotic bacteria, cgl caudal gland, de ductus ejaculatorius, gb gubernaculum, na non-annulated tip of tail, s sperm, sp spiculum, vel velum).

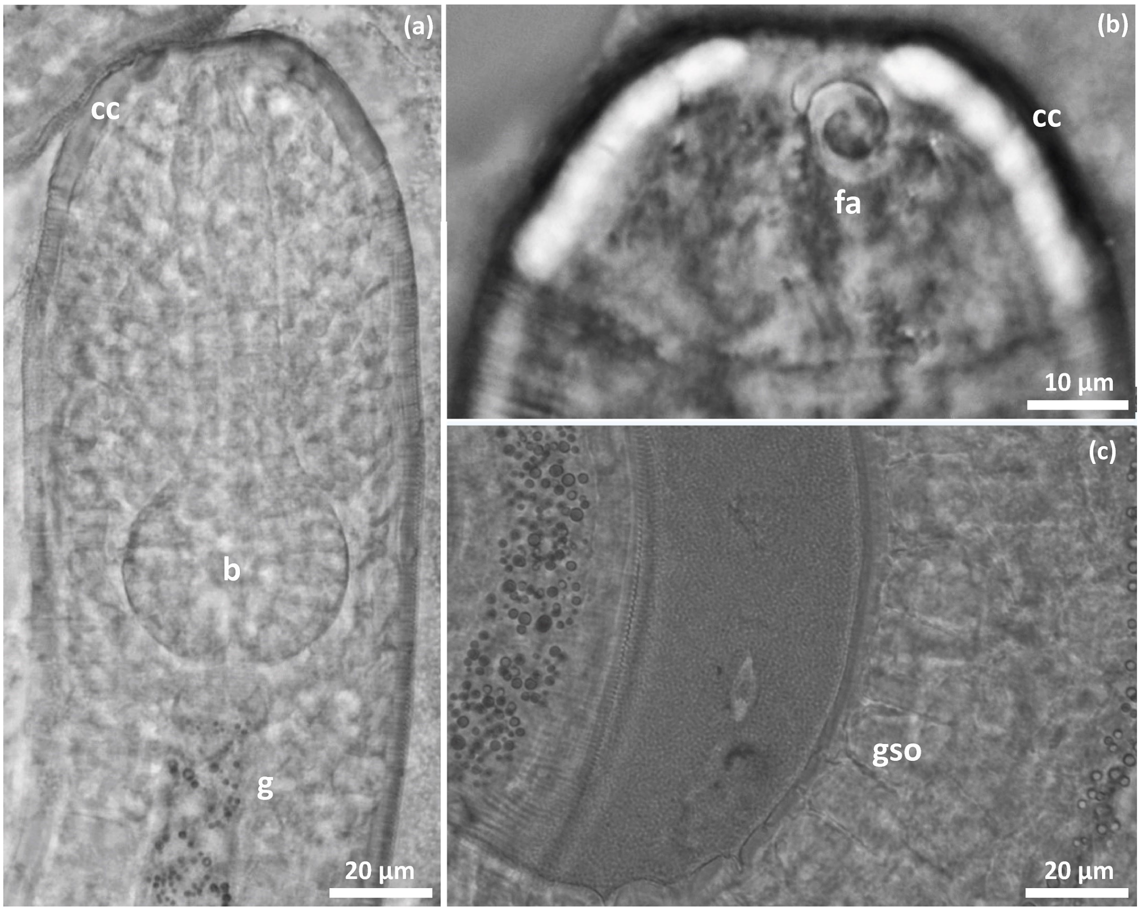

Fig. S6. Light micrographs of preserved specimen of *Paralaxus* sp. ``heron 1´´ (a) anterior region (b) anterior end (c) midbody region with dissociated bacterial coat (b pharyngeal bulbus, cc cephalic capsule, fa fovea amphidalis, g gut, gso glandular sense organ).

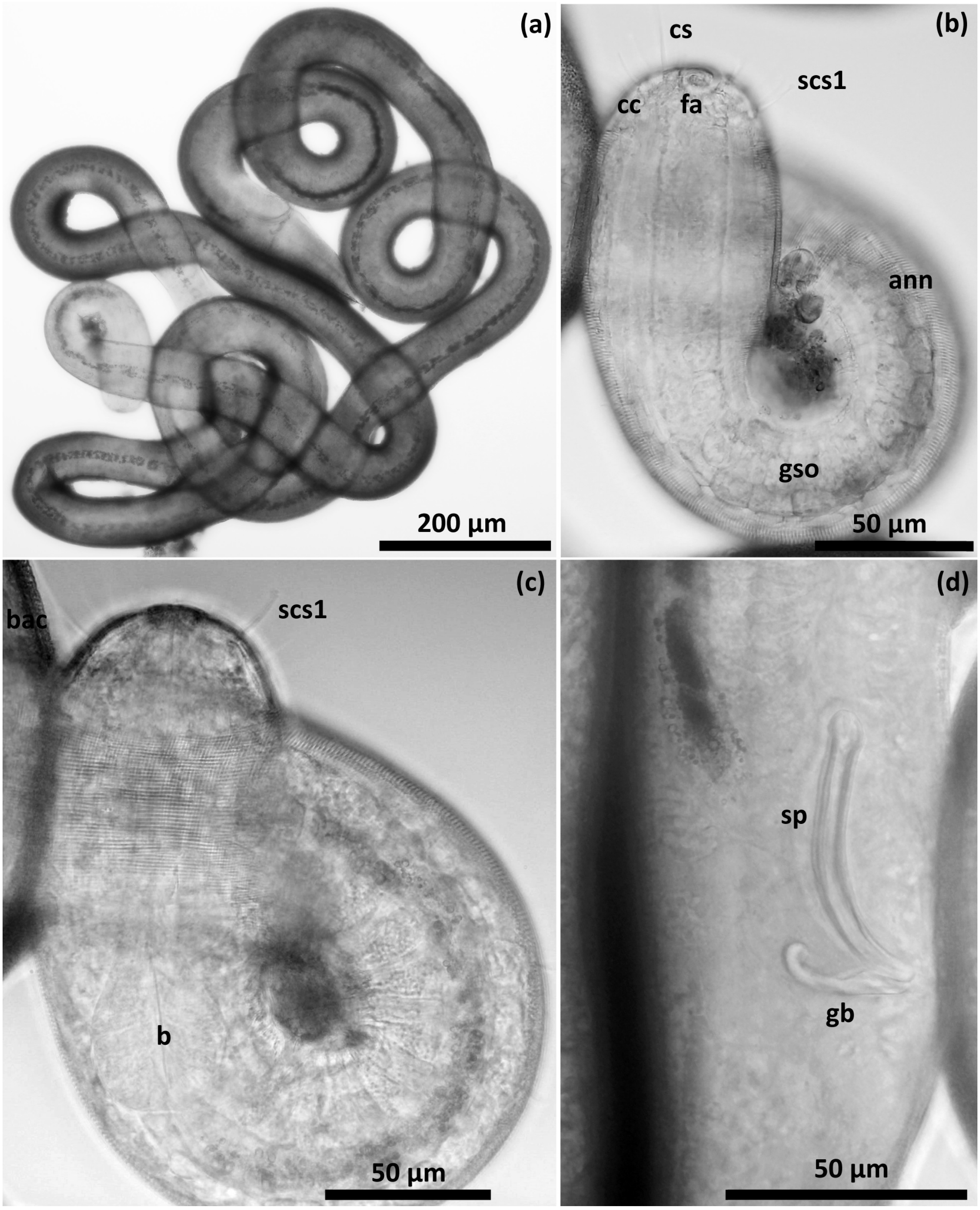

Fig. S7. Light micrographs of live specimen of *Paralaxus* ``oahu 1´´ male. (a) total view (b) anterior region (c) optical section of anterior region (d) cloacal region (ann annulation, b pharyngeal bulbus, bac bacteria, cc cephalic capsule, cs cephalic setae, gb gubernaculum, gso glandular sense organ, scs1 subcephalic setae of first circle, sp spiculum).

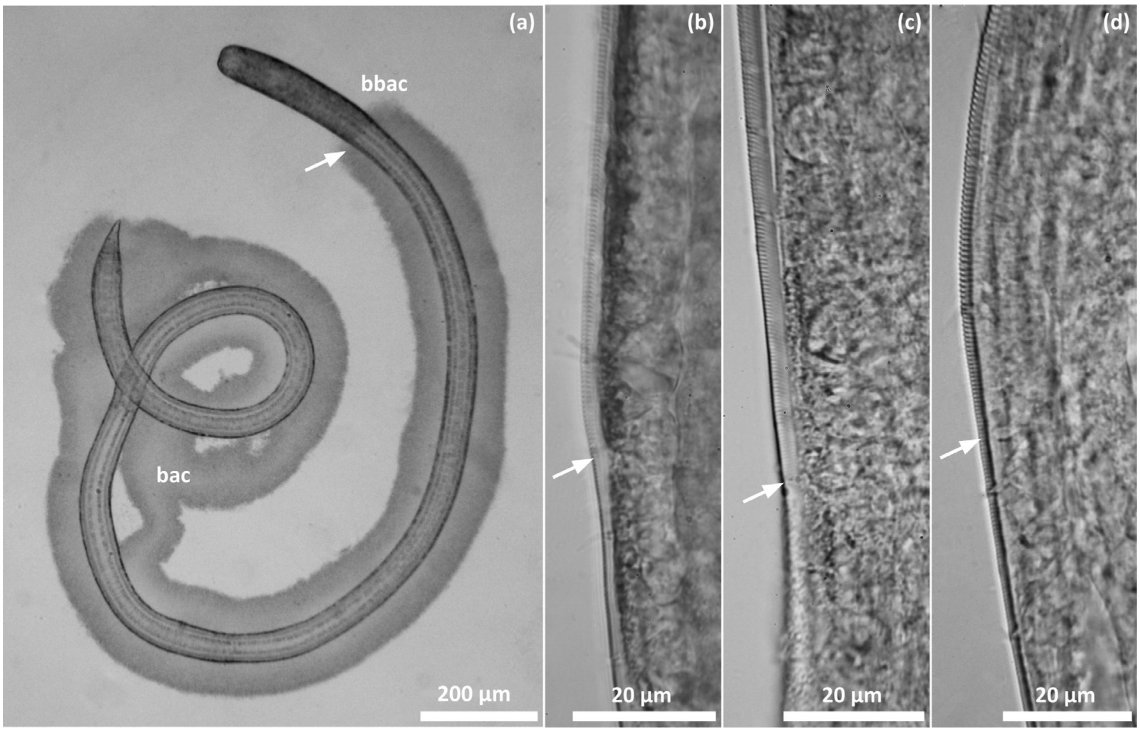

Fig. S8. Light micrographs of preserved specimen of *Paralaxus* species and its morphological adaptation to its symbiont coat. (a) *Paralaxus columbae* sp. nov., juvenile. Bacterial coat starting at the transition from coarse to finer annulation; (b – d) Transition in adult specimens; (b) *P. cocos* sp. nov. (c) *P. columbae* sp. nov. (d) *P. bermudensis* sp. nov. The transition points are marked by white arrows (bac bacteria, bbac beginning of bacterial coat).

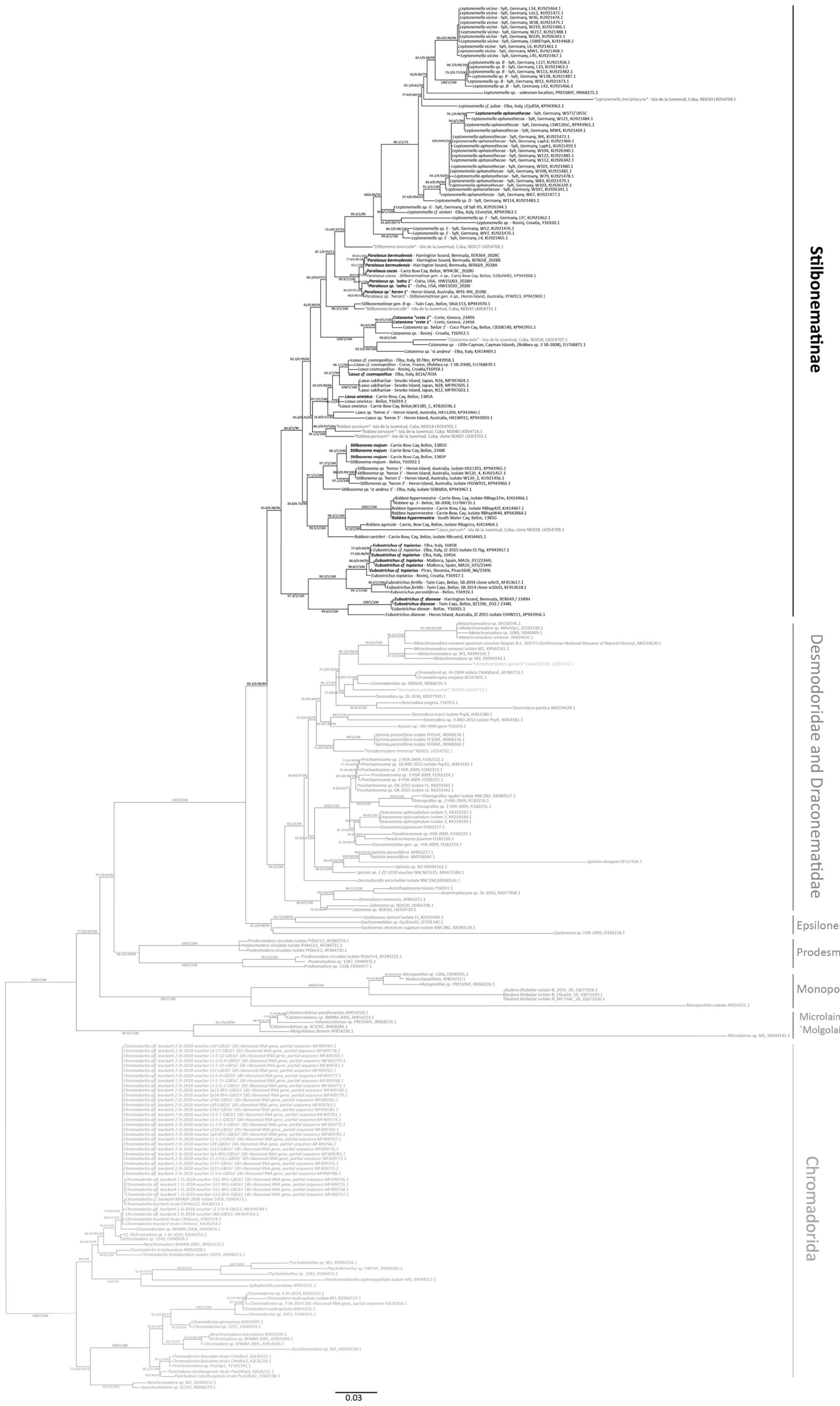
Stilbonematinae
Desmodoridae and Draconematidae
Epsilonematidae
Prodesmodorinae
Monoposthiidae
Microlaimidae and `Molgolaimus´
Chromadorida
0.03

Fig. S9. Host 18S rRNA phylogeny of extended dataset (non-collapsed tree version)

The tree was calculated using IQ-Tree. Support values are given in the following order: SH-aLRT support (%) / aBayes support/ ultrafast bootstrap support (%). Species described in this study are highlighted in bold. Species from Armenteros et al. (2014b) are highlighted in italic and light grey, with quote marks. Provisional working names for undescribed genera or species are given in quotes. The scale bar represents average nucleotide substitutions per site.

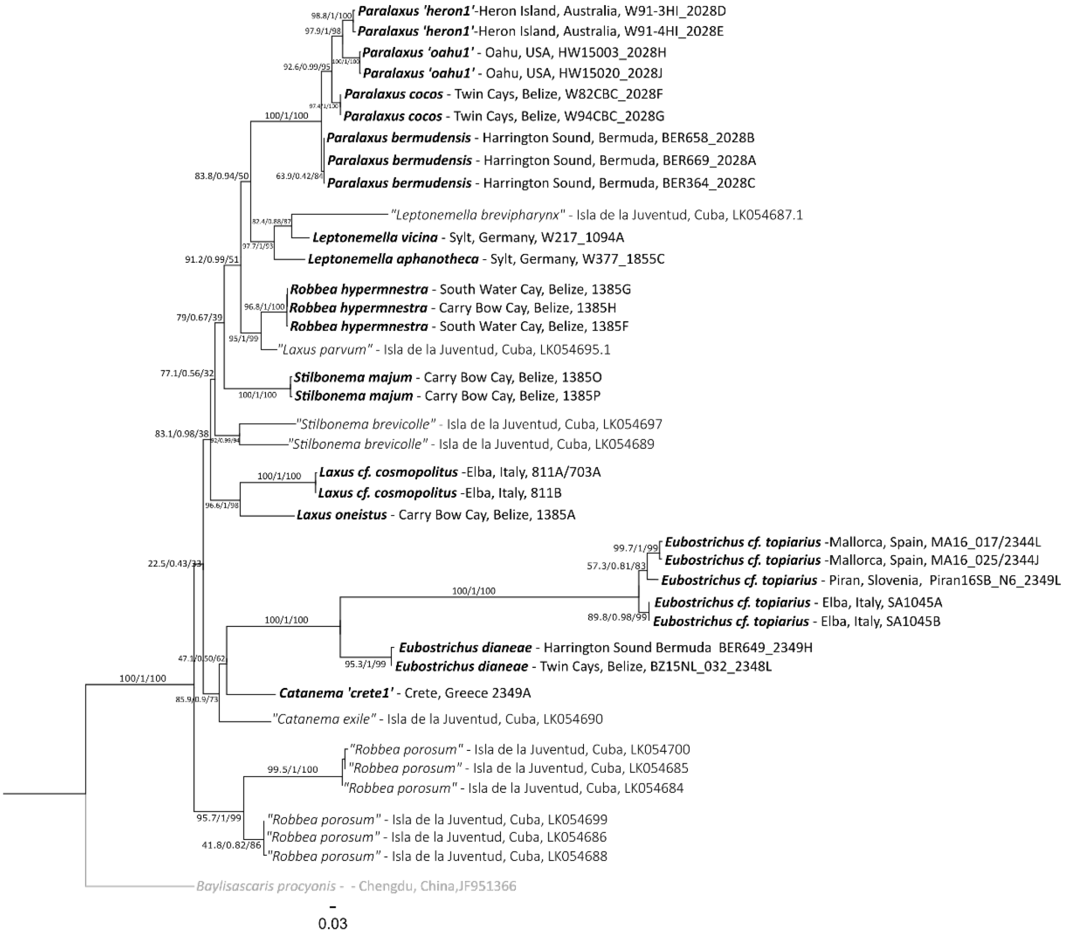

Fig. S10. Host COI phylogeny of extended dataset (non-collapsed tree version)

The tree was calculated using IQ-Tree. Support values are given in the following order: SH-aLRT support (%) / aBayes support/ ultrafast bootstrap support (%). Species described in this study are highlighted in bold. Species from Armenteros et al. (2014b) are highlighted in italic and with quote marks. Outgroup in light grey and italic. Provisional working names for undescribed genera or species are given in quotes. The scale bar represents average nucleotide substitutions per site.

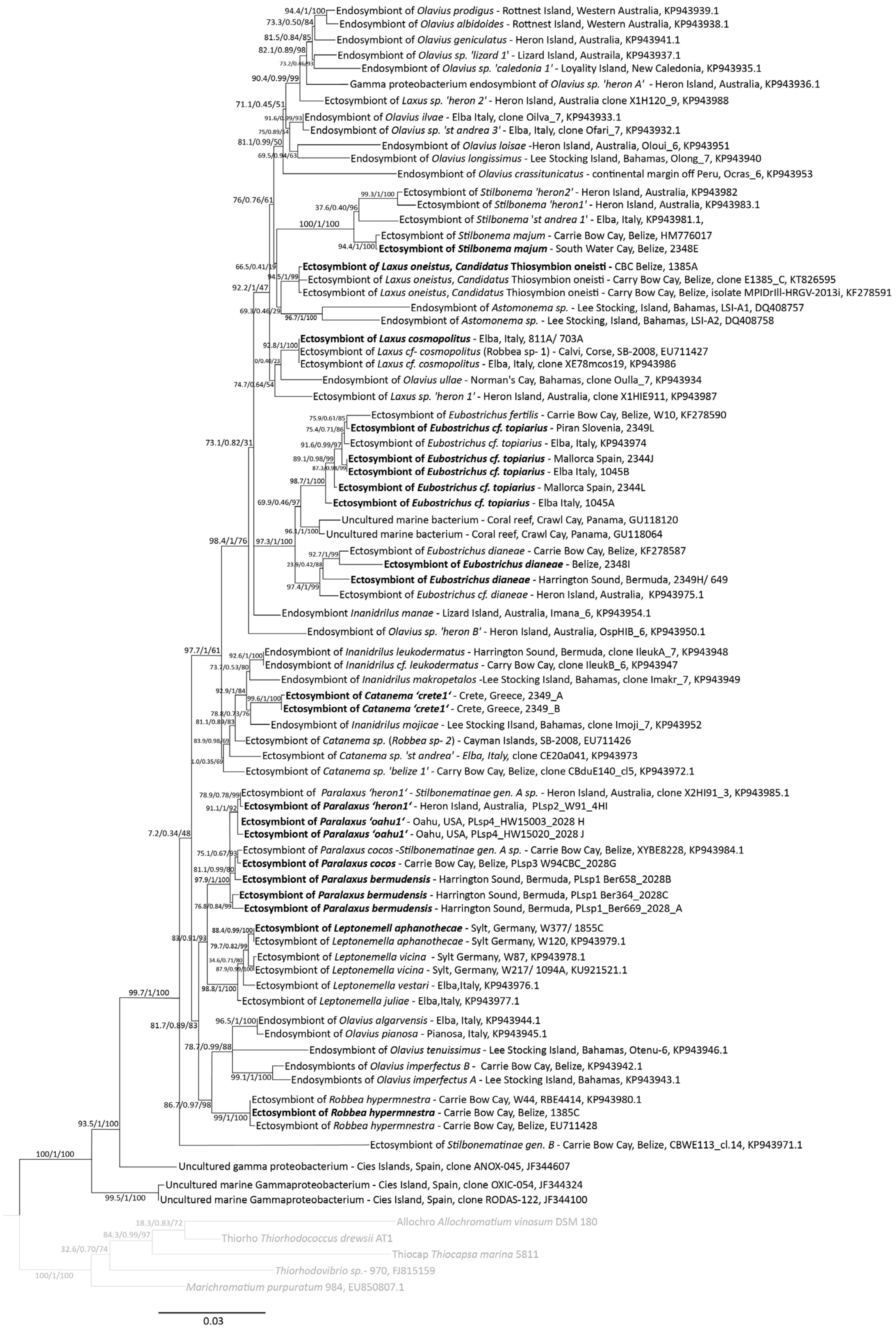

Fig. S11. Symbiont 16S rRNA phylogeny (non-collapsed tree version)

The tree was calculated using IQ-Tree. Support values are given in the following order: SH-aLRT support (%) / aBayes support/ ultrafast bootstrap support (%). Symbiont sequences generated in this study are highlighted in bold. Outgroup in light grey. Provisional working names for undescribed genera or species are given in quotes. The scale bar represents average nucleotide substitutions per site.
